# Multi-ancestry Transcriptome-Wide Association Study Reveals Shared and Population-Specific Genetic Effects in Alzheimer’s Disease

**DOI:** 10.1101/2025.11.03.686160

**Published:** 2025-11-05

**Authors:** Xinyu Sun, Makaela Mews, Nicholas R. Wheeler, Penelope Benchek, Tianjie Gu, Lissette Gomez, Nicholas Ray, Christiane Reitz, Adam C. Naj, Jennifer Elizabeth Below, Giuseppe Tosto, Mario Cornejo-Olivas, Goldie S. Byrd, Briseida E. Feliciano-Astacio, Katrina Celis, Farid Rajabli, Brian W. Kunkle, Margaret A. Pericak-Vance, Jonathan L. Haines, Anthony J. Griswold, William S. Bush

**Affiliations:** 1 Department of Population and Quantitative Health Sciences, School of Medicine, Case Western Reserve University, Cleveland, Ohio, USA.; 2 System Biology & Bioinformatics; Department of Nutrition, School of Medicine, Case Western Reserve University, Cleveland, Ohio, USA.; 3 Cleveland Institute for Computational Biology, Case Western Reserve University, Cleveland, Ohio, USA.; 4 Taub Institute for Research on Alzheimer’s Disease and the Aging Brain, Vagelos College of Physicians and Surgeons, Columbia University, New York, New York, USA.; 5 The Gertrude H. Sergievsky Center, Vagelos College of Physicians and Surgeons, Columbia University, New York, New York, USA.; 6 Department of Neurology, Vagelos College of Physicians and Surgeons, Columbia University, and the New York Presbyterian Hospital, New York, New York, USA.; 7 Department of Biostatistics and Epidemiology, University of Pennsylvania Perelman School of Medicine, Philadelphia, Pennsylvania, USA.; 8 Penn Neurodegeneration Genomics Center, Department of Pathology and Laboratory Medicine, Perelman School of Medicine, University of Pennsylvania, Philadelphia, Pennsylvania, USA.; 9 Vanderbilt University Medical Center; 10 Neurogenetics Working Group, Universidad Cientifica del Sur, Lima, Peru.; 11 Neurogenetics Research Center, Instituto Nacional de Ciencias Neurologicas, Lima, Peru.; 12 Maya Angelou Center for Health Equity, Wake Forest University, Winston-Salem, North Carolina, USA.; 13 Universidad Central del Caribe, Bayamón, Puerto Rico.; 14 John P. Hussman Institute for Human Genomics, Miller School of Medicine, University of Miami, Miami, Florida, USA; 15 Dr. John T. Macdonald Foundation Department of Human Genetics, University of Miami Miller School of Medicine, Miami, Florida, USA

## Abstract

Alzheimer’s disease (AD) risk differs across ancestral populations, yet most genetic studies have focused on Non-Hispanic White (NHW) cohorts. We conducted a multi-population transcriptome-wide association study (TWAS) using whole-blood RNA-seq and genotype data from reported NHW (n=235), African American (AA; n=224), and Hispanic (HISP; n=292) participants in MAGENTA. Using SuShiE for multi-population fine-mapping, we identified credible sets of eQTLs for 8,748 genes and improved fine-mapping precision relative to analyses using fewer populations. eQTL effects were largely shared across populations, with population-specific regulation for a subset of genes. Population-stratified TWAS and sample size–weighted meta-analysis (FUSION + MAFOCUS) prioritized and and fine-mapped nine genes (FDR<0.05, PIP>0.8), including established AD loci (*BIN1*, *PTK2B*, *DMPK*) with consistent effects across populations. Importantly, at *BIN1* we fine-mapped regulatory variants associated with gene expression and AD risk beyond the GWAS index SNP—most notably rs11682128, which is only in modest LD with rs6733839 ($r^{\wedge }2\approx 0.34$)—demonstrating that multi-population TWAS can implicate additional functional variants not captured by single-SNP GWAS signals. We also discovered a novel association between COG4 expression and AD in NHW, implicating Golgi apparatus function. Using independent SuShiE-derived models from TOPMed MESA (PBMC), several associations replicated directionally across ancestries, with statistical significance most evident in NHW. Our results show that multi-population fine-mapping improves eQTL resolution and TWAS interpretability, reveals regulatory variants beyond GWAS index SNPs, and underscores the need to expand non-European AD cohorts to resolve shared and population-specific mechanisms.

## Introduction

Alzheimer’s Disease (AD) is the most common form of dementia and affects millions worldwide, with its prevalence expected to increase significantly as global populations age^1^. AD is heritable, though AD risk varies substantially across ancestral populations, with African Americans having approximately double the risk and Hispanics having about one and one-half times the risk compared to non-Hispanic Whites^1,2^. Recent and ongoing ascertainment efforts have increased participation of African American and Admixed Hispanic individuals in genetic studies, which have led to multiple ancestry-specific GWAS^3^, and a recent multi-ancestry GWAS meta-analysis^4^. These studies (along with detailed analyses of APOE- 4 effects^5^) have revealed substantial heterogeneity in effect sizes of established AD risk variants^4^, and different genetic architectures in AD risk.

Growing recognition that AD involves coordinated dysregulation of both peripheral and central immune systems has shifted the field toward viewing AD as a systemic disorder. As highlighted by Bettcher et al.^6^, mounting clinical and mechanistic evidence reveals dynamic crosstalk between the peripheral immune system and the central nervous system, suggesting that immune perturbations in blood may not only reflect, but also influence, AD pathogenesis. This systems-level perspective underscores the value of studying whole blood as an informative, accessible tissue for understanding disease mechanisms across diverse populations.

Multiple transcriptome-wide association studies (TWAS) of AD have been conducted to extend GWAS results to functional effects in brain and blood^7–17^. In principle, TWAS accelerates the advancement of interventions by jointly modeling associations from multiple (typically non-coding) variants to estimate the effect of changes in gene expression on a phenotype, which provides more biologically interpretable relationships than single variant associations alone. Various multi-variable modeling strategies (such as Elastic net^18^ and LASSO^19^) have demonstrated gene expression is predominantly driven by a sparse set of genetic variants^20–22^. Recent methodological advances in Bayesian fine mapping^23,24^ have also led to approaches that use fine-mapped cis-eQTLs as variables for gene expression prediction^21,25^. Bayesian fine-mapping methods such as SuSIE quantify uncertainty by defining credible sets of highly correlated variants, which enables more robust interpretation in the presence of complex linkage disequilibrium (LD) structures^23^. Notably, all of these approaches require population-specific eQTL reference panels^26^ that have distinct eQTL effects specific to a particular population and also matches the LD structure of the target GWAS dataset. Due to the scarcity of non-European eQTL datasets, nearly all TWAS have been conducted in Non-Hispanic white populations only.

In this work, we perform multi-ancestry fine-mapping of whole-blood eQTL effects and conduct the first multi-ancestry TWAS of AD within reported African American (AA), Non-Hispanic White (NHW), and Hispanic (HISP) participants in the MAGENTA study. We first build population-specific gene expression prediction models; then perform population-stratified TWAS, integrate these associations via sample-size–weighted meta-analysis, and fine-map putative causal genes. Finally, we compare dense (all cis-SNPs) versus sparse (credible set–based) models. We report novel association signals and further dissect the blood-based functional genetic architecture of known AD association signals.

## Methods

### eQTL Reference Panel Study Participants

The *Multi-Ancestry Genomics, Epigenomics, and Transcriptomics of Alzheimer’s* (MAGENTA) study accessed existing sample collections that were part of multiple previous NIA-funded research projects. Some legacy samples were drawn from studies that ascertained White or African American participants. All other samples were drawn from more recent studies including the Puerto Rican Alzheimer’s Disease Initiative, the Cuban American Alzheimer’s Disease Initiative, the Peru Alzheimer’s Disease Initiative, and the Research in African-American Alzheimer’s Disease Initiative. All participants and/or their consenting proxy provided written informed consent as part of the study protocols approved by the site-specific Institutional Review Boards.

### Population Descriptors and Study Group Definitions

Consistent with recent recommendations^27^, we define our rationale and definition for the population descriptors in this study. MAGENTA participants were recruited across multiple studies that differed in catchment geography (U.S., Peru), terms used for study recruitment and outreach, and cultural context. As a result, demographic fields and participant descriptors varied across individuals.

To enable cross-group comparisons while acknowledging differences in genetic ancestry and admixture, we used a combination of geographic and self-identity terms as follows:

- “Non-Hispanic White” (NHW): individuals ascertained in North Carolina, Tennessee, and South Florida where participants self-identified as Non-Hispanic White or were categorized as not Hispanic in legacy datasets where recontact is impossible.
- “African American” (AA): individuals ascertained in Northeast Ohio, North Carolina, and South Florida using descriptors intended to reach self-identified Black/African American participants.
- “Caribbean Hispanic”: individuals ascertained in the U.S. using descriptors intended to reach individuals with Cuban or Puerto Rican heritage, reflecting similar admixture patterns and environmental exposures.
- “Peruvian Hispanic”: individuals ascertained in Lima, Peru and surrounding areas.

For consistency with prior published GWAS studies, we combined the Caribbean Hispanic and Peruvian Hispanic into a single “Hispanic” group to facilitate our TWAS analyses. These labels reflect recruitment context and self-identification; downstream genetic analyses incorporate ancestry-informative covariates to account for population structure.

### Genotyping and Quality Control

All samples were genotyped using either the Illumina MEGA v2 or v3 multi-population array and subsequently imputed using TOPMed reference panels v2 (build 38) corresponding with self-identified ancestry and/or ascertainment criteria. We restricted our analysis to variants directly genotyped by the array and variants with an imputation quality control score of $R^{2}>0.8$. Genotyping data was processed using the ADSP xqtl-protocol release 0.1.1. All analyses were performed stratified by group yielding a total of three analyses. Relatedness was assessed using kinship analysis generated by the KING software. Subsequently, we removed samples to eliminate relatedness (kinship coefficient ≥ 0.0625) and maintained samples by prioritizing inclusion of cognitively normal individuals with advanced age, diagnosed AD cases, and control samples with rarer APOE genotypes (ε3/4 or ε4/4). We concluded our genotyping quality control using PLINK2.0 to filter variants with minor allele counts (MAC) of 10, Hardy-Weinberg equilibrium of 1 * 10^−8^, variant-level missingness of 0.1, and sample-level missingness of 0.1.

Post-QC, 224 AA, 235 NHW, 292 HISP samples from MAGENTA, with both whole-blood expression and genotype data, were analyzed. The gene expression was measured in whole blood using RNA-seq. We included the common protein-coding genes that were available in all three populations. In total, we retained 14,436 genes. Major Histocompatibility Complex (MHC) region from chromosome 6p (~24–34 Mbp) was excluded from the study, due to the complex LD structure. In the end, we analyzed 14,211 protein-coding genes.

### RNA Processing

Sample preparation and RNA sequencing of whole blood samples have been previously described^28^. Briefly, RNA was extracted from previously frozen whole blood samples, quality controlled with RNA integrity (RIN) scores >= 5, prepared using the NuGEN Universal Plus mRNA-Seq with globin and ribosomal depletion, and sequenced using 125bp paired end Illumina HiSeq3000. RNA sequencing FASTQ files were processed using the STAR aligner (v.2.7.10a), quality controlled using Picard, and quantified using RSEM (v.1.3.3) using the following reference files: GRCh38_full_analysis_set_plus_decoy_hla.noALT_noHLA_noDecoy_ERCC.fast a, Homo_sapiens.GRCh38.103.chr.reformatted.collapse_only.gene.ERCC.gtf, and /hg38_GENCODE.v38.bed (see ADSP xqtl-protocol 0.1.1 for full details). Genes with a TPM expression level of 10% or less in over 20% of samples and/or genes with read counts less than 6 among fewer than 20% of samples were excluded from the analysis. In addition, samples detected as outliers using relative log expression (RLE), hierarchical clustering, and D-statistics were removed. Finally, TPM data was normalized using the Trimmed Mean of M-value (TMM) method.

### Bulk RNA-seq Deconvolution and Hidden Factor Detection

As we used bulk whole blood RNA data for our analysis, we performed CIBERSORTx deconvolution using the European-based LM22 reference panel to estimate relative cell-type proportions in each sample^29^. We performed this analysis using all samples combined. We included estimated cell-type proportions for 14 estimated cell types as covariates in our analysis based on an identified significant difference in relative cell-type proportions within each population or across AD status (Kruskal-Wallis Test p < 0.05). The 14 estimated cell-type proportions are Neutrophils, CD4+ T cells-Memory resting, B cells – Naïve, B cells – Memory, Macrophages – M1, Mast cells – activated, T cells – follicular helper, Plasma cells, T cells – CD4 naive, Regulatory T cells – Tregs, Macrophages – M0, NK cells – activated, Monocytes, and NK cells - resting.

We also performed Hidden Factor Analysis using the Marchenko-Pastur PCA approach as described in the ADSP xqtl-protocol 0.1.1. The approach applies the Marchenko-Pastur distribution to determine the optimal number of principal components to retain by modeling the eigenvalues of random covariance matrices. Components with eigenvalues above the Marchenko-Pastur threshold are considered to represent meaningful biological variation, while those below it are attributed to random noise. This technique ensures that only components capturing substantive biological variation are retained for downstream analyses, avoiding overfitting while enhancing the interpretability of confounder correction in our QTL mapping.

### AD GWAS Summary Statistics

We accessed the largest available clinically diagnosed AD GWAS summary statistics for each population. All summary statistics were derived from models adjusted for age, sex, and population substructure principal components. We lifted coordinates from GRCh37/hg19 using UCSC liftOver^30^ as needed.

We accessed summary statistics from the Ray et al. African American GWAS, which included 2,784 AD cases and 5,222 controls (total n=9,168)^31^. We also accessed summary statistics from the Hispanic subset of Rajabli et al., comprising 3,005 AD cases and 5,894 controls (total n=8,899)^4^. For the Non-Hispanic White population, we used Stage I data from Kunkle et al., which included 21,982 AD cases and 44,944 controls (total n=63,926)^32^.

### Harmonization of GWAS and Expression Prediction Reference

To ensure consistency and improve the integration of GWAS summary statistics and our gene expression prediction reference panel, we performed several harmonization procedures. First, we ensured the reference and alternative alleles of each variant were defined consistently across the GWAS and the reference panel. If the reference and alternative alleles were flipped between the GWAS and the reference panel, we inverted the GWAS Z-score so that its sign matched the alternative allele in the reference panel. Second, since the prediction model prioritizes weights mostly to eQTL variants within credible sets, we performed summary statistics imputation to maximize the power and SNP coverage. We followed the PredictDB GTEx v8 gwas imputation pipeline to impute the missing variant z-scores in GWAS summary statistics, according to the MAGENTA genotype reference panel for each population. 1,252,104, 1,253,727, and 1,081,547 variants are imputed for AA, EUR, and HISP GWAS respectively, which represented 19.07% of total variants on average. Third, only unambiguous SNPs, that do not involve substitutions of complementary base pairs, were kept.

### Statistical methods

#### SuShiE

SuShiE^33^ incorporated individual level gene expression and genotype data from all three populations to infer credible sets and population-specific weights. We followed empirical convention^23,24,33^ and selected the expected maximum number of credible sets, L, as 10 for each protein-coding gene. We specified posterior inclusion probability (PIP) threshold for SNPs to be included in the credible sets as 0.9. In some cases, credible sets may contain a few lead variants in tight LD and low LD to others in a region. By definition, each credible set captures a homogeneous and distinct signal. To refine the final inference results, credible sets with lowest absolute pairwise correlation, which is defined as *purity*, and weighted by sample sizes across all populations, are removed, if the purity is less than 0.5. For the remaining parameters, default settings are used (Table S2).

SuShiE produces population-specific cis-eQTL weights to predict gene expression (GReX). SNPs with large magnitude weights were identified by SuShiE as members of credible sets. Such variable selection approaches reduce TWAS estimation errors by reducing the potential of weak gene expression prediction instruments^34^. SuShiE also computed cis-SNP heritability using the limix python package for each gene within each population.

Cis-eQTL weighted prediction modeling was performed for each protein-coding gene using SNPs within a ± 500kb region, adjusting for age, sex, PCs, cell-type proportions and hidden factors.

#### FUSION

FUSION estimates the association between genetically regulated gene expression (GReX) and Alzheimer’s disease using GWAS summary statistics. The TWAS test statistic ($Z_{\mathrm{TWAS}}$) is computed as a weighted combination of GWAS SNP-level Z-scores ($Z_{\mathrm{gwas}}$), where the weights ($W_{\mathrm{snp}}$) are derived from the SNPs’ effects on gene expression. To account for correlation structure among SNPs, the test statistic is normalized by the square root of the weighted linkage disequilibrium (LD) matrix:

$$Z_{\mathrm{TWAS}}=\frac{W_{snp}*Z_{\mathrm{gwas}}}{\sqrt{W_{snp}*LD*W_{snp}}}$$

We followed the FUSION^35^ pipeline and used MAGENTA genotype data as the LD reference panel for each population-stratified TWAS analysis. There are 14,127, 14,116, and 13,742 gene models available for NHW, AA, HISP respectively. There are 13,731 common models shared across all populations. SuShiE-derived population-specific cis-eQTL effect sizes were used to predict the gene expression for each gene, and identify potential AD-related genes for each population.

#### Meta analysis of population-stratified TWAS associations

Effective sample size weighting (SSW) meta-analysis was performed on three population-stratified TWAS associations to share TWAS associations across all populations. While inverse-variance weighting (IVW)^26^ is generally preferred, summary-level TWAS methods like FUSION cannot produce the effect sizes (beta) and their standard errors required for this approach.

Due to the limited correlations and concordances of TWAS associations between pairs of two populations, that was similarly observed in prior studies^26^, we set the FDR’s q-value threshold to 0.05 ($q_{c}<0.05$), in order to identify shared AD-related gene candidates.

### TWAS Fine-mapping

TWAS would nominate multiple genes, while some genes could be non-causal due to sharing of eQTLs or co-regulated genes. We applied MAFOCUS^36^ to prioritize putative causal genes by correcting for correlation structures induced by linkage disequilibrium (LD) and prediction weights in our TWAS results. Population-specific genotype data from NHW, AA, and HISP groups in the MAGENTA study served as LD reference panels. We performed fine-mapping on LD blocks containing at least one significant gene ($q_{c}<0.05$) using default parameters. For each LD block, MAFOCUS calculated posterior inclusion probabilities (PIPs) and derived 80% credible sets (ρ=0.8) of putative causal genes, both for individual populations and across all three populations. To prioritize genes with the strongest evidence of causality, we established PIP thresholds based on the MAFOCUS methodology; we classified a significant TWAS association as a high-confidence causal gene if it achieved either a population-specific $PIP_{\mathrm{pop}}>0.8$ in its corresponding population-stratified analysis, or a multi-population $PIP_{ME}>0.8$ in the sample-size weighted meta-analysis that integrated evidence across all populations.

### Independent Multi-ancestry Expression Reference Panel

In our analyses, we re-evaluate the significant TWAS associations from our discovery dataset (MAGENTA) using the publicly released SuShiE-derived weights from the TOPMed MESA visit-1 mRNA dataset^33,37,38^. As we did not have access to the individual level genotype of the MESA samples, we used high-coverage 1000 Genomes data to generate a LD reference for each ancestry^39^. The MESA dataset contained expression measurements from 956 participants (402 European American, 175 African American, and 277 Hispanic American individuals) with corresponding whole genome sequencing data. The mRNA expression was quantified in peripheral blood mononuclear cells (PBMCs), covering 21,747 protein-coding genes. The original analysis adjusted for 15 gene expression principal components, 10 genotype principal components, age, sex, and assay lab as covariates. These SuShiE-derived prediction models were accessed at https://zenodo.org/records/10963034.

## Results

### MAGENTA Study Overview

We used the Multi-Ancestry Genomics, Epigenomics, and Transcriptomics of Alzheimer’s (MAGENTA) cohort as the eQTL reference, comprised of African American (AA, n=224), Non-Hispanic White (NHW, n=235), and a combined Hispanic group (HISP, n=292). The Hispanic group contains Caribbean and Peruvian sub-cohorts to align with available AD GWAS summary statistics. Case/control status was approximately balanced across groups (~50% each), and age distributions were comparable (means ~76.6–77.5 years), supporting cross-population comparability for downstream fine-mapping and TWAS analyses (Table 1).

We found that cis-eQTL effects were largely consistent whether or not Alzheimer’s disease (AD) status was included as a covariate in the MAGENTA datasets (Figure S3, Table S3, Figure S4), consistent with previous studies^40–42^.

### From multi-ancestry fine-mapping to interpretable TWAS: workflow overview

**Figure 1:**
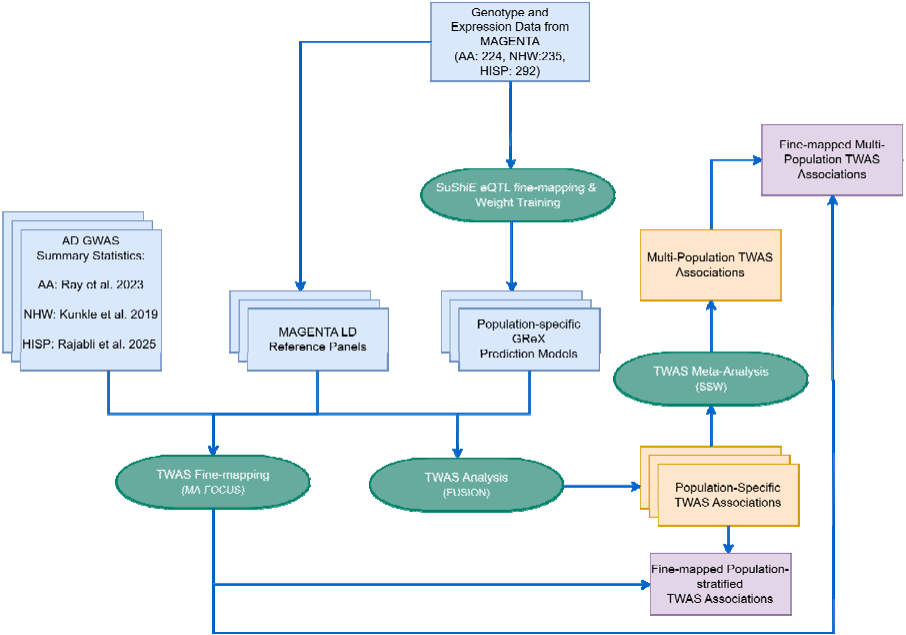
Multi-population TWAS analytical workflow for Alzheimer’s Disease. The workflow integrates MAGENTA genotype and expression data (n=751; AA: 224, NHW: 235, HISP: 292) with published AD GWAS summary statistics across three population groups: Hispanic (Rajabli et al. 2023), African American (Ray et al. 2022), and Non-Hispanic White (Kunkle et al. 2019). SuShiE is applied for multi-population eQTL fine-mapping to create population-specific GReX prediction models, which are then used in FUSION analysis alongside GWAS summary statistics and population-matched reference panels. Population-stratified TWAS results are combined through sample size weighted (SSW) meta-analysis to produce multi-population TWAS associations. Fine-mapping of these results using MA-FOCUS identifies the most likely causal genes.

Our multi-population workflow (Figure 1) integrates MAGENTA expression and genotype data with population-matched AD GWAS to generate interpretable TWAS associations. SuShiE multi-ancestry fine-mapping yields population-specific GReX models that we test with FUSION within each ancestry and meta-analyzed across populations, followed by MAFOCUS gene-level fine-mapping of TWAS hits to identify putative causal genes. A detailed dense versus sparse comparison demonstrating that credible-set variants recapitulate dense-model TWAS signals is presented in the “Sparse Models Using Fine-mapped eQTLs” subsection. Overall, We showed this workflow increases eQTL resolution and enables variant-resolved interpretation of TWAS signals.

### Multi-population fine-mapping improves credible set precision and identifies shared eQTL effects

Using MAGENTA whole-blood RNA-seq and genotypes from AA, NHW, and HISP (14,436 shared protein-coding genes), we applied SuShiE multi-ancestry fine-mapping and identified credible sets (CS) for 8,748 genes. The mean number of CS per gene was 1.27 (median 1), with very few genes requiring more than seven CSs, supporting our choice of L=10. Credible sets were compact (median 4 SNPs; Figure S1), consistent with a sparse cis-regulatory architecture^20–22,43,44^.

Including all three populations improved fine-mapping precision relative to any two-population analysis (NHW–AA, NHW–HISP, and AA–HISP) (Table 2); median SNPs per CS decreased from 6 to 4 (−33%), consistent with reduced LD-induced ambiguity across ancestries, the mean number of CS per gene increased from 0.52 to 1.27 (+144%), and the fraction of SNPs with PIP>0.9 rose from 0.018 to 0.024 (+32%), indicating improved confidence in variant-level fine-mapping. These findings are consistent with the benefits of multi-ancestry fine-mapping results reported by the PAGE consortium^45^.

**Figure 2:**
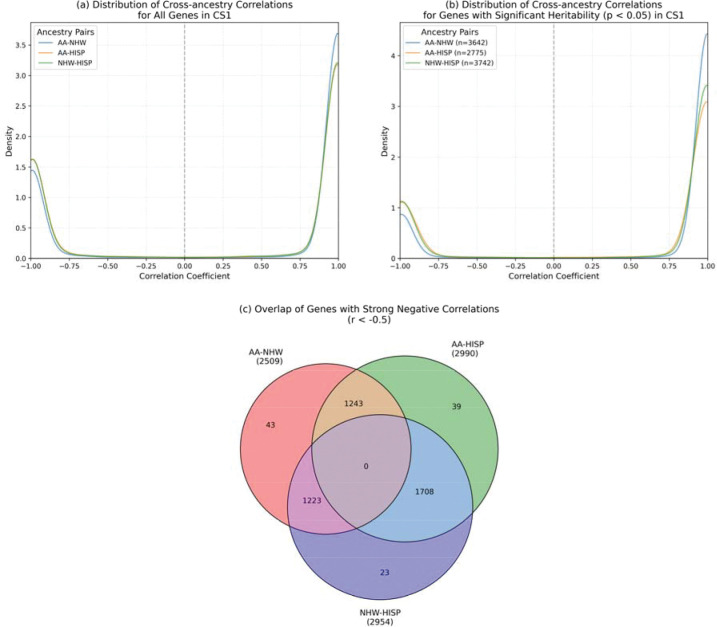
Cross-population analysis of eQTL effects in the first credible set (CS1). (a) Density plots showing the distribution of effect size correlations across population pairs (NHW-AA, NHW-HISP, and AA-HISP) for all genes (n=8,748) (left) and genes with significant heritability (right, p < 0.05). The bimodal distributions indicate predominantly consistent effects across populations (~70% positive correlations) with some population-specific patterns. (b) Venn diagram showing overlap of genes with strong negative correlations (r<−0.5) between population pairs. The substantial pairwise overlaps (1223–1708 genes) suggests that genetic effects typically differ in one population while remaining consistent between the other two, with HISP showing the most distinct patterns (1708 overlapping genes with other pairs).

Following the approach of Lu et al.^33^, we evaluated cross-ancestry consistency of effects for first SuShiE reported single shared effect (CS1), which represented the largest effects on gene expressions. Effect size correlations were highly consistent for roughly 70% of genes, indicating broadly shared regulation (Figure 2) across groups. A subset of genes (~30%) showed anticorrelation (r<−0.5), with 2,509 (AA–NHW), 2,990 (AA–HISP), and 2,954 (NHW–HISP) genes, and substantial pairwise overlap (1,223–1,708 genes) where genes have a different direction of effect in one population compared to the other two. These findings highlight population-specific signals with HISP showing the most distinct patterns.

We then assessed posterior eQTL weight concordance of variants in CS1 across populations. For all genes, correlations were moderate (AA–NHW r=0.526; AA–HISP r=0.379; NHW–HISP r=0.441), and increased when restricting to genes with significant cis-heritability (AA–NHW r=0.689; AA–HISP r=0.484; NHW–HISP r=0.540) (Figure S2). These results support substantial sharing of cis-regulatory mechanisms across ancestries, with clearer concordance in genes under stronger genetic control though notable population-specific effects remain.

### Population-stratified TWAS reveals both shared and group-specific AD gene associations

**Figure 3:**
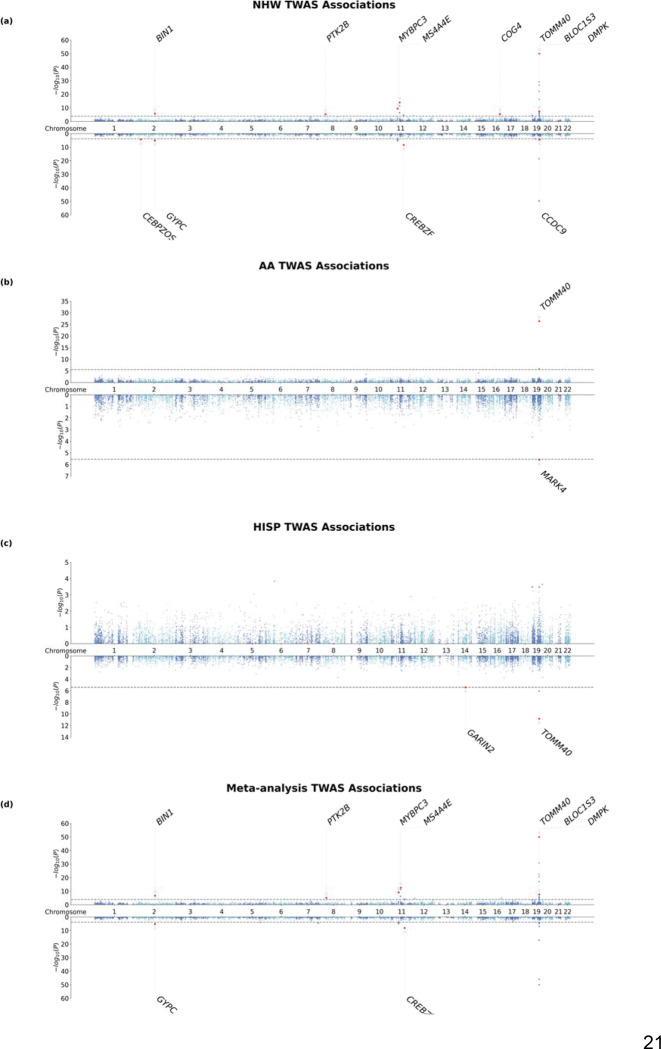
population-stratified and meta-analyzed TWAS associations for Alzheimer’s Disease across diverse populations. The figure displays TWAS associations as Manhattan plots with −log10(P-value) on the Y-axis, with positive associations shown in the top panel and negative associations in the bottom panel. The gray dashed lines indicate the population-stratified FDR threshold of 0.05, corresponding to $P_{NHW}<1.4\;*\;10^{-4};\;P_{AA}<2.8\;*\;10^{-6};\;P_{\mathrm{HISP}}<3.8\;*\;10^{-6};\;P_{\mathrm{META}}<1.4\;*\;10^{-4}$ for each respective population. Red dots highlight genes that meet both statistical significance (FDR<0.05) and fine-mapping criteria (PIP>0.8), with gene symbols labeled for these prioritized associations. Each panel represents different population-stratified or meta-analyzed associations. a) A Miami plot of NHW population-stratified TWAS associations; b) A Miami plot of AA ancestry-population TWAS associations; c) A Miami plot of HISP population-stratified TWAS associations; d) A Miami plot of SSW meta-analyzed TWAS associations

We applied MAGENTA-derived, population-specific weights in FUSION to AD GWAS from Kunkle et al. (NHW), Ray et al. (AA), and Rajabli et al. (HISP). Test calibration was acceptable ($\lambda_{GC}$: NHW = 1.080, AA = 0.909, HISP = 0.979). At $q_{c}<0.05$ (NHW: $P<1.4\;*\;10^{-4}$; AA: $P<2.8\;*\;10^{-6}$; HISP: $P<3.8\;*\;10^{-6}$), we identified 40, 4, and 3 significant genes, respectively (Figure 3 (a–c); Table S6). Using MAFOCUS to prioritize putative causal genes among the significant hits $\left(q_{c}<0.05\right)$, we identified high-confidence genes with PIP>0.8: 12 in NHW and 2 each in AA and HISP.

We first highlight within-population signals ($q_{c}<0.05$, population-specific PIP>0.8) that did not reach significance in the multi-population meta-analysis. In NHW, two genes outside known AD loci were significant: *CEBPZOS* and *COG4* (Figure 3 (a)). *CEBPZOS* showed a negative association ($Z_{NHW}=-4.076,\;P=4.58\;*\;10^{-5}$). SuShiE fine-mapping identified 11 SNPs in 5 credible sets; CS1–CS2 exhibited near-perfect cross-population effect concordance ($\overset{‾}{\rho }\approx \;0.999$), whereas CS3–CS5 were population-specific (anticorrelated). In particular, NHW’s effect size correlation from CS4–5 exhibited strong anticorrelation than HISP and AA (Figure S5). Expression was strongly predictable in NHW ($R^{2}=\;0.225$) and modestly but significantly in AA ($R^{2}=0.0246,\;P\;=0.0106$). Using independent SuShiE models from TOPMed MESA, the NHW effect direction validated ($Z_{NHW}=-2.513$). No genome-wide significant GWAS SNPs were present within ±1 Mb ($P<5\;*\;10^{-8}$; the most significant association is from rs1072218 $P=5.50\;*\;10^{-5}$). The direction agrees with prior NHW TWAS^46^ (OR=0.97; 0.96–0.99).

*COG4* showed a significant association with AD risk in NHW ($q_{c}<0.05,\;Z_{\mathrm{NHW}}=\;4.601,\;P=4.20\;*\;10^{-6}$; population-specific PIP>0.8). SuShiE fine-mapping identified 9 SNPs in two credible sets, with strong anticorrelation of effects between NHW and the other populations ($\overset{‾}{\rho }\approx -0.934$). Expression was genetically predictable in both NHW ($R^{2}=0.0487,\;P<0.05$) and AA ($R^{2}=0.123,\;P<0.05$), yet AA showed no TWAS association ($Z_{AA}=-0.00317,\;P=0.997$). No genome-wide significant GWAS SNPs were present within ±1 Mb ($P<5\;*\;10^{-8}$; the most significant association is from rs3752786 $P=3.98\;*\;10^{-6}$). Using independent SuShiE models from TOPMed MESA, the NHW direction replicated but was not significant ($Z_{NHW}=0.281,\;P=0.779\}$); attenuation is plausibly due to reduced cis-SNP coverage and differing fine-mapped eQTLs in MESA (±500 kb; MAGENTA $N_{snp}=1358$ vs. MESA $N_{snp}=783$, −42.3%) and potential age/AD-context differences.

In HISP TWAS, one gene reached significance at $q_{c}<0.05$: *GARIN2* (also known as *FAM71D*) on 14q23.3 ($Z_{\mathrm{HISP}}=-4.622,\;P=3.80\;*\;10^{-6}$) (Figure 3 (c)). No SuShiE credible sets were identified and 5-fold CV was not significant ($R^{2}=-0.00345,\;P=0.991$); SuShiE posterior weights in HISP were uniformly small ($max\;magnitude\approx \;1\;*\;10^{-5}$), indicating the TWAS signal is unlikely to be mediated by predicted expression. The result may instead reflect aggregated weak SNP effects within HISP that may tag other variants in the region (Figure S8).

In AA TWAS, *MARK4* was significant ($Z_{AA}=-4.710,\;P=2.50\times 10^{-6}$) within the *APOE* region (~ 170,000 bp from *APOE*). SuShiE fine-mapping identified 13 SNPs across five credible sets; CS1 showed near-perfect cross-ancestry concordance (${\overset{‾}{\rho }}_{1}\approx 1$), while averaging across all sets indicated heterogeneity (${\overset{‾}{\rho }}_{AA-NHW}\approx 0.200$; ${\overset{‾}{\rho }}_{AA-HISP}\approx 0.599$; ${\overset{‾}{\rho }}_{NHW-HISP}\approx -0.199$). Predictive performance was not significant in AA (5-fold CV $R^{2}$ not significant ($R^{2}=0.0033;\;P=0.190=$)), but was significant in NHW and HISP ($R_{NHW}^{2}=0.308;\;P=1.35\;*\;10^{-20},\;R_{\mathrm{HISP}}^{2}=0.102;\;P=1.52\;*\;10^{-8}$). In TOPMed MESA, the AA effect direction was consistent but not significant ($Z_{AA}=-1.662,\;P=9.66\;*\;10^{-2}$). Given regional complexity and proximity to *APOE* (Figure S7), this association should be interpreted with caution.

We explored shared TWAS associations using Sample Size Weighted (SSW) Meta-analysis. There are 39 statistically significant (${q_{c}<0.05;P_{\mathrm{META}}<1.4\;*\;10}^{-4}$) genes, and 9 of them had MAFOCUS’s PIP>0.8 (Figure 3(d)). All fine-mapped meta-analysis associations were driven mostly by the NHW population, due to its larger sample size. Relative to the NHW TWAS, no new associations became significant after meta-analysis. However, the sign concordance of the 9 identified genes between NHW and the other two populations is 66.67%, which demonstrates moderate consistency and some heterogeneity across populations.

In total, we identified multiple significant TWAS associations across chromosomes 2, 8, 11, and 19, with variable patterns of genetic regulation across populations (Table Table 3).

### Chromosome 2 – *BIN1* and *GYPC*

*BIN1* showed highly consistent TWAS associations across AA, NHW, and HISP populations, with fine-mapping implicating three SNPs in two credible sets (mean cross-population effect size correlation $\overset{‾}{\rho }\approx 0.956$). The lead eQTL rs11682128 (previously linked to *BIN1* expression in blood and brain) was in moderate LD ($r^{2}\approx 0.34$; 53,336 bp away) with the top GWAS SNP rs6733839. 5-fold cross-validation $R^{2}$ (0.116–0.263) was significant in all populations.

*GYPC*, located near *BIN1*, also showed consistent TWAS associations but had significant $R^{2}$ only in the HISP population.

### Chromosome 8 – *PTK2B*

*PTK2B* was significant in the meta-analysis and fine-mapping identified 28 SNPs in three credible sets with high effect size concordance ($\overset{‾}{\rho }\approx 0.995$). Notably, the 28 SNPs are in minimal LD with the GWAS index SNP, rs2741342, with the highest LD in the first credible set ($r^{2}\approx 0.1$). Predictive performance ($R^{2}=0.040-0.120$) were significant in all populations, though TWAS significance was observed mainly in NHW, with consistent direction across datasets.

### Chromosome 11 – *MYBPC3*, *MS4A4E*, and *CREBZF*

*MYBPC3* showed the strongest signal in NHW, with high cross-population effect size correlation $\left(\overset{‾}{\rho }\approx 0.999\right)$. $R^{2}$ was significant in only NHW ($h^{2}=0.052$). *MS4A4E* was driven by the NHW signal, with weak or negative effect size correlation with AA and HISP (AA-NHW: ρ≈0.004; AA-HISP: ρ≈−0.004) and no significant predictive performance in any population. *CREBZF* showed no significant $R^{2}$, or fine-mapped variants, and was likely influenced by nearby strong GWAS signals ($P<1\;*\;10^{-8}$ from 86Mb to 86.2Mb).

### Chromosome 19 – *TOMM40*, *DMPK*, and *BLOC1S3*

Among the three genes near APOE locus, only *TOMM40*’s SuShiE TWAS prediction model contained APOE-ε4 allele, rs429358 in its first CS.

*TOMM40* exhibited strong but heterogeneous associations, with significant $R^{2}$ only in NHW and variable effect directions across populations. The learned prior effect size correlations showed substantial heterogeneity across populations especially in HISP, with moderate positive correlation between AA and NHW ($\overset{‾}{\rho }\approx 0.599$), but negative correlations between AA-HISP ($\overset{‾}{\rho }\approx -0.492$) and NHW-HISP ($\overset{‾}{\rho }\approx -0.190$). *DMPK* showed consistent positive direction across populations, moderate cross-population correlations $\left({\overset{‾}{\rho }}_{\mathrm{AA-HISP}}\approx 0.661\right.;\;{\overset{‾}{\rho }}_{AA-NHW}\approx 0.382;\;{\overset{‾}{\rho }}_{NHW-HISP}\approx 0.053$), and significant predictive performance ($R^{2}=0.026-0.146$) in all groups. *BLOC1S3* had highly consistent effect sizes across populations ($\overset{‾}{\rho }\approx 0.998$) but significant $R^{2}$ only in NHW $\left(R^{2}=0.031\right.;\;h^{2}=0.048$).

Across loci, patterns emerged where some genes (*BIN1*, *PTK2B*, *BLOC1S3*) had consistent cross-population genetic regulation, whereas others (*MYBPC3*, *TOMM40*, *MS4A4E*) displayed marked population-specific architectures in one group. Using direction as the primary criterion, we observed replicated effects in TOPMed MESA for several discovery genes in at least two ancestries—notably *BIN1*, *PTK2B*, *DMPK*, *TOMM40*, and *MYBPC3*, and *MS4A4E*—with significance most evident in NHW and generally insignificant in AA and HISP, consistent with GWAS sample size differences. Detailed association results, cis-$h^{2}$, $R^{2}$, and replication outcomes are presented in Table 3, Table S5, and Table S7.

### Sparse models using fine-mapped eQTLs capture key TWAS associations and implicate variants beyond GWAS index SNPs

Across fine-mapped TWAS hits (MAFOCUS PIP>0.8), credible sets typically did not include the GWAS index SNPs. At *BIN1*, the top CS SNP rs11682128 was only modestly correlated with the NHW index SNP rs6733839 ($r^{2}\approx 0.34$), indicating additional regulatory variants beyond the GWAS lead. Similar patterns were observed at *PTK2B* and *DMPK*, where rs2741342 and rs429358 are not in the credible sets for each gene respectively. However, in the APOE region *TOMM40*’s CS1 included the ε4-defining rs429358 and drove heterogeneous associations across populations. Dense-versus-sparse comparisons showed that restricting models to credible-set variants largely preserved association magnitudes (Table 4), supporting a fine-mapped variant-level regulatory basis for these TWAS signals.

To confirm that fine-mapped eQTLs in credible sets provide adequate models for TWAS associations, we compared the TWAS Z-scores between two approaches: a dense model using SuShiE expression estimates for all SNPs within the cis-region (±500kb), and a sparse model using only SNPs identified in credible sets. Our analysis focused on significant genes identified in the dense model approach, examining the consistency and magnitude of associations across both modeling strategies. A detailed summary of the credible sets for these significant genes, including the number of variants, posterior inclusion probabilities, and other fine-mapping statistics, is provided in the Supplementary Material (Table S4).

The comparison revealed remarkable consistency between dense and sparse models for most genes (Table 4). Key AD-associated genes like *BIN1*, *TOMM40*, *COG4*, and *MARK4* showed particularly stable associations across both approaches. For instance, *BIN1* maintained consistent negative associations across all populations in dense and sparse models (NHW: 4.73 vs 4.34; AA: 2.06 vs 2.00; HISP: 1.86 vs 1.60), with only slight attenuation in the sparse model. Similarly, *TOMM40* preserved its strong population-specific pattern, showing nearly identical Z-scores in both approaches for all populations (NHW: 47.7 vs 48.3; AA: 10.8 vs 10.8; HISP: −6.75 vs −6.88). However, the strong and heterogeneous associations of *TOMM40* are likely driven by the APOE effect, as the APOE ε4 allele, rs429358, is present in the first CS.

The novel association we identified, *COG4*, also demonstrated stability between dense and sparse models (NHW: 4.6 vs 3.88; AA: 0 vs 0.29; HISP: −0.93 vs −0.42), suggesting that its association with AD is primarily driven by the fine-mapped eQTLs.

However, some genes showed notable differences between the two approaches. For example, *BLOC1S3* in the APOE region showed different magnitudes of association in the dense versus sparse models in the NHW population (21.2 vs 3.47). This difference could be explained by the complex LD structure and APOE signals in the region, where many variants could contribute to the association signal through LD with known AD risk variants. Similarly, *GYPC* showed marked differences in the NHW population (−4.43 vs 0.06), suggesting that some of its association might be driven by SNPs outside the credible sets. Since *GYPC* is in close proximity to *BIN1*, the strong TWAS association of *GYPC* in NHW, is likely to be driven by rs6733839 (not in CSs of *GYPC*), which is an index SNP in the region of NHW AD GWAS.

Overall, our comparison demonstrates that fine-mapped eQTLs in credible sets can largely explain the TWAS associations for most genes, particularly those with strong and consistent effects across populations. The stability of associations between dense and sparse models provides additional support for the sparse genetic architecture of gene expression regulation and validates our fine-mapping approach.

**Figure 4:**
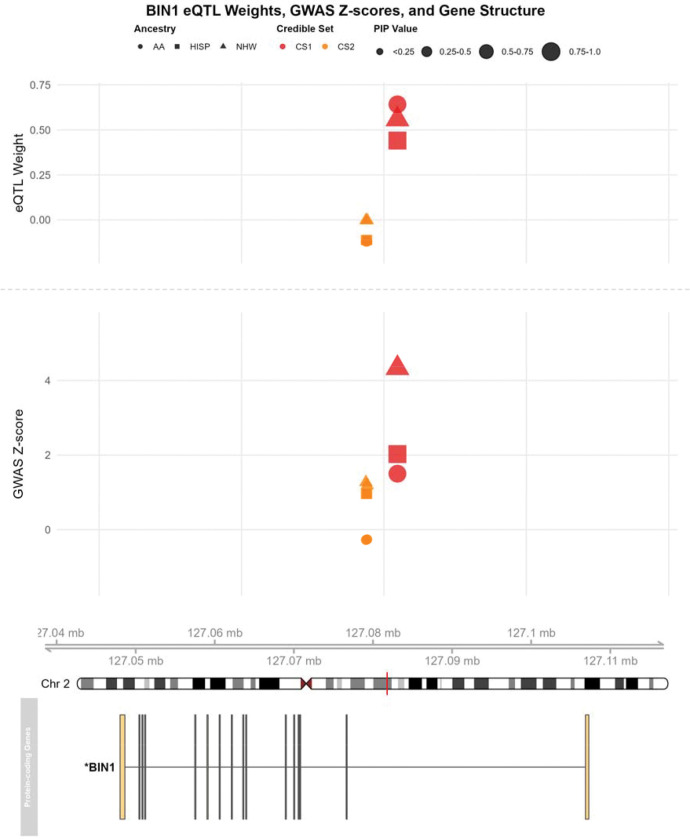
Fine-mapped eQTLs and GWAS associations for the BIN1 locus. The figure displays the relationship between eQTL weights (top panel), GWAS Z-scores (middle panel), and gene structure (bottom panel) for BIN1 on chromosome 2. In the top and middle panel, eQTL weights and GWAS associations are shown across three ancestral populations (African American (AA, circles), Hispanic (HISP, squares), and Non-Hispanic White (NHW, triangles)), with variants colored by credible set membership. Point sizes indicate posterior inclusion probability (PIP) values. The bottom panel illustrates the genomic structure of BIN1 at chromosome 2. In CS1, there is only one variant, rs11682128; In CS2, there are two variants, rs6714626 and rs6710752

As illustrated in Figure 4, *BIN1*’s variant (rs11682128) in the first credible set (CS1) exhibited consistently positive eQTL weights across all three populations, with magnitudes ranging from 0.44 to 0.64. The visualization further revealed population-specific patterns in the corresponding GWAS signals, with the strongest Z-scores observed in the NHW population (~4.5), followed by more moderate signals in AA and Hispanic populations (1–2), which may explain the magnitude of TWAS associations across ancestries. The difference could be due to the sample size differences in GWAS studies, or real effect size differences across populations as we observe with APOE^5^. Despite these differences in GWAS signal intensity, the direction and relative magnitude of the TWAS associations remain consistent (Table 4), suggesting that the identified credible set variants captured the core regulatory mechanisms influencing BIN1 expression in relation to AD risk. The genomic context provided in the bottom panel demonstrates that the two credible sets are located in the intronic region of *BIN1*. Collectively, this integrated view of BIN1 regulation supports our finding that sparse models using only credible set variants can effectively recapitulate the signals detected in dense models, while providing greater interpretability regarding the specific variants driving these associations.

**Figure 5:**
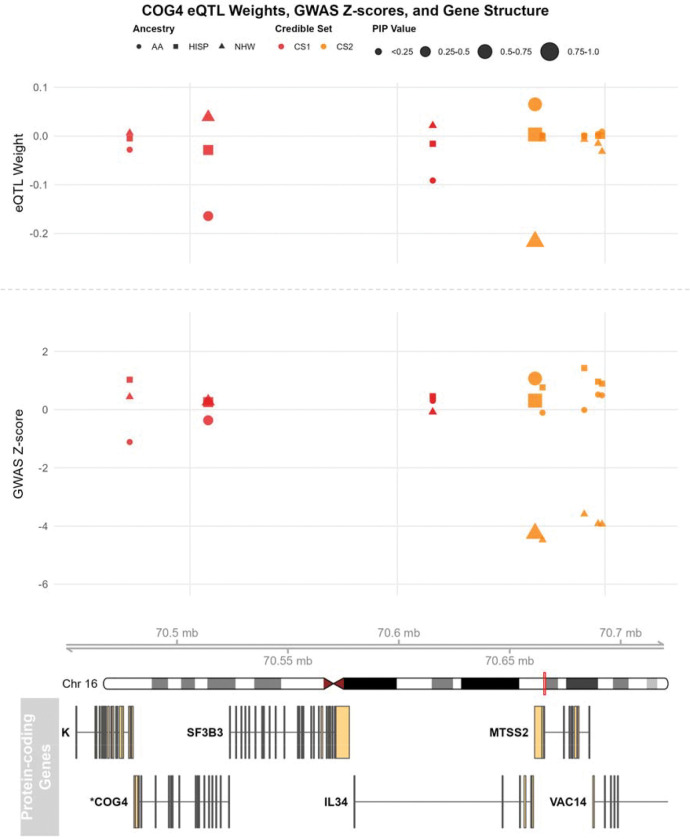
Fine-mapped eQTLs and GWAS associations for the COG4 locus. The figure displays the relationship between eQTL weights (top panel), GWAS Z-scores (middle panel), and gene structure (bottom panel) for TOMM40 on chromosome 19. In the top and middle panels, eQTL weights and GWAS associations are shown across three ancestral populations (African American (AA, circles), Hispanic (HISP, squares), and Non-Hispanic White (NHW, triangles)), with variants colored by credible set membership. Point sizes indicate posterior inclusion probability (PIP) values. The bottom panel illustrates the genomic structure of COG4 at chromosome 16.

*COG4* is identified as a novel AD-related gene from our main analysis. When we focused on the sparse model, which included only 9 variants in 2 credible sets, we observed a similar TWAS association ($Z_{NHW}=3.88$) in the NHW population, while the other two populations did not show significant associations (Table 4). After analyzing the SNP weights and GWAS associations for these fine-mapped SNPs (Figure 5), we noticed substantial differences in the NHW population, particularly in the second credible set. For example, rs11639579, the fine-mapped SNP in the second credible set with the largest PIP (PIP=0.709), exhibited the largest difference in NHW’s GWAS association and SNP weight compared to the other two populations (Table 4). We observed that this variant is also located near *IL34*, but the SNP is not fine-mapped in *IL34*’s credible set, and the TWAS association of *IL34* is not significant in the dense model.

## Discussion

In this multi-ancestry TWAS of AD, we show that jointly modeling cis-eQTLs across African American, Non-Hispanic White, and Hispanic groups improves fine-mapping resolution and yields more interpretable, variant-resolved associations. Through the lens of whole-blood gene expression, we implicate regulatory variants beyond GWAS index SNPs at established loci at BIN1, PTK2B, and DMPK. We further nominate COG4 as a novel candidate gene for AD.

Importantly, we demonstrate that sparse credible-set models from our fine-mapping recapitulate dense-model associations, concentrating evidence onto compact variant sets without a significant loss of association signal. Our multi-ancestry fine-mapping of eQTL results decreased the number of SNPs in each credible set while also increasing the overall number of credible sets. These findings are consistent with the results reported in Lu et al.^33^. and provide additional empirical evidence supporting the sparse genetic architecture of gene expression regulation previously reported in multiple studies^20–22^. Our results also align with prior studies showing that while differences in LD and allele frequencies across ancestral groups might lead to heterogeneous eQTL effects, the correlation is largely consistent across populations^33,47–49^.

Our multi-population TWAS analysis revealed both known and novel associations with AD risk. Importantly, many of the TWAS associations appear to be driven not by GWAS index SNPs, but by fine-mapped regulatory variants highlighted in credible sets, suggesting that the detected AD TWAS associations are driven by subsets of gene expression regulatory variants. We identified regulatory variants for *BIN1*, *PTK2B*, *DMPK*, and *GYPC*, all mostly independent of established GWAS signals at these loci. *BIN1* demonstrated remarkable consistency in TWAS associations that were successfully confirmed in the TOPMed MESA dataset across three groups. Importantly, our results confirmed the eQTL effect of rs11682128 is shared across three populations, which was previously reported by Zhu et al^50^. Lastly, two additional SNPs were identified in the second credible set, which implied more regulatory variants in BIN1.

We also identified *CEBPZOS* (which has been reported from prior TWAS^50^) and *COG4*, neither of which have nearby genome-wide significant GWAS signals. To the best of our knowledge, *COG4* had not been identified as an AD-related gene previously. Notably, single-ancestry fine-mapping in NHW alone identified no credible sets, whereas including all three populations yielded two credible sets, underscoring the advantage of multi-ancestry fine-mapping for variable selection. Specifically, four variants in the first credible set had higher allele frequency in AA than the other two populations Table S8. *COG4* encodes a component of an oligomeric protein complex that plays a crucial role in the structure and function of the Golgi apparatus, which is essential for processing and distributing proteins necessary for neuronal function. Notably, rs11639579, the SNP in the second credible set with the largest PIP (PIP=0.709), exhibited substantial differences in GWAS association and SNP weight in the NHW population compared to AA and HISP populations. According to Agora^51^, an AD knowledge portal, *COG4* has meaningful expression across brain regions. The differential expression, between AD cases and controls, is significant in cerebellum (${log}_{2}FC=-0.149,\;P=1.44\;*\;10^{-3}$) and parahippocampal gyrus (${log}_{2}FC=0.0827,\;P=1.44\;*\;10^{-2}$). Furthermore, Golgi fragmentation occurs in many neurodegenerative diseases. In AD, Golgi fragmentation results in enhanced APP trafficking and Aβ production^52,53^. The significant association of genetically predicted *COG4* expression in blood with AD suggests a potentially underexplored link between systemic vesicle trafficking pathways and disease risk.

Our study faces several important limitations. First, while whole blood is readily accessible and often used in large-scale genomic studies, it may not optimally reflect the tissue-specific regulatory mechanisms occurring in the brain. Currently, the scarcity of brain-specific eQTL data from diverse populations constrains our ability to conduct optimal TWAS analysis and validate these findings in the most relevant tissue. However, several previous literatures observed largely concordant AD TWAS effect size directions and eQTL effect sizes between brain and peripheral tissues^54,55^. Additionally, Huseby et al. found eight fundamental cell biological functions that are altered in whole blood samples from six neurodegenerative diseases^56^, suggesting AD risk can be modulated by multiple systemic factors^57^. Second, the substantial disparity in GWAS sample sizes between populations significantly impacted our ability to detect population-specific associations. This imbalance is reflected in our meta-analysis results, where the significant associations were predominantly driven by NHW signals, as evidenced by the stronger Z-scores in NHW population across most identified genes (Table 3). Nevertheless, despite these power limitations, we observed consistent effect directions across populations for several key genes. Third, a practical limitation of multi-ancestry fine-mapping (including SuShiE) is the requirement to analyze only SNPs shared across populations to ensure matched LD and comparability; such filtering can exclude ancestry-unique variants, biasing credible sets toward shared signals and potentially underestimating truly population-specific regulatory variants, especially in AA and admixed HISP where allele frequencies and LD differ. We attempted to mitigate extreme sparsity by applying a per-population MAC threshold (MAC ≥ 10). Future work should explicitly accommodate ancestry-unique variants in multi-ancestry fine-mapping method developments, and expand recruitment of underrepresented populations to enable adequately powered ancestry-specific fine-mapping and TWAS that are comparable to EUR-based studies. Forth, we acknowledge that reliance on the European-derived LM22 panel may reduce cell type deconvolution accuracy and introduce population-specific bias in our AA and HISP cohorts, so these cell-type covariates should be interpreted cautiously and revisited with ancestry-matched or single-cell–derived references as they become available.

Several key future directions could build upon our findings. First, as brain-specific multi-population eQTL datasets become available, replicating our analyses in neural tissue would be valuable for validating these associations and potentially uncovering additional AD-related genes. More critically, ongoing efforts to expand AD GWAS in non-European populations will be crucial for achieving more balanced statistical power across populations. This enhanced power would not only improve our ability to detect population-specific effects but also provide more robust evidence for shared genetic mechanisms across populations. Such advancements would help address the current European bias in genetic studies and contribute to a more comprehensive understanding of AD’s genetic architecture across diverse populations.

## Supplementary Material

Supplement 1

## Figures and Tables

**Table 1:** Sample characteristics of MAGENTA study participants.

	Subcate gory	All	AA	NHW	HISP

Sample Size (N)		751	224	235	292
Sex	Male	267 (35.6%)	64 (28.6%)	110 (46.8%)	93 (31.8%)
	Female	484 (64.4%)	160 (71.4%)	125 (53.2%)	199 (68.2%)
Age (years)	Mean ± SD	77.0 ± 7.7	76.6 ± 7.8	77.5 ± 7.4	76.9 ± 7.9
	Median [IQR]	77.0 [71.0–83.0]	76.5 [70.0–83.0]	78.0 [72.0–83.0]	77.0 [71.0–83.0]
	Range	53.0–98.0	63.0–97.0	65.0–97.0	53.0–98.0
Status	Case	378 (50.3%)	111 (49.6%)	118 (50.2%)	149 (51.0%)
	Control	373 (49.7%)	113 (50.4%)	117 (49.8%)	143 (49.0%)

* The table presents demographic and clinical characteristics of the MAGENTA study across all participants and stratified by population groups (AA: African American, NHW: Non-Hispanic White, HISP: Hispanic). For each characteristic, counts and percentages are shown for categorical variables, while mean ± standard deviation, median [interquartile range], and range are provided for continuous variables. Sample sizes, sex distribution, age statistics, and Alzheimer’s Disease status are reported for the total cohort and each population group.

**Table 2:** Improved fine-mapping precision of shared cis-eQTLs through multi-population analysis.

Metric	Average of Any Two Populations	Three Populations (NHW+AA+HISP)	Improvement

Median SNPs per credible set	6	4	33% reduction
Mean credible sets per gene	0.52	1.27	144% increase
Percent of eQTL had PIP > 0.9	0.018	0.024	32% increase

* This table demonstrates the benefits of incorporating three diverse populations (Non-Hispanic White, African American, and Hispanic) in SuShiE analysis compared to using only two populations. The tri-population approach resulted in more precise credible sets (33% fewer SNPs per set), identified more credible sets per gene (144% increase), and increased the proportion of SNPs with high posterior inclusion probability (32% increase in SNPs with *PIP* > 0.9). These findings support the sparse genetic architecture of gene expression regulation and highlight the value of diverse population inclusion in fine-mapping studies.

**Table 3:** Multi-population TWAS significant genes identified by SSWmetaanalysis.

CHR	SBP	EBP	Gene Symbol	Meta Z	Meta P	Z AA	Z NHW	Z HISP

2	126656133	126696667	GYPC	−4.555	5.242e-06	−1.213	−4.433	−0.292
2	127048027	127107288	BIN1	5.176	2.269e-07	2.056	4.734	1.856
8	27311482	27459391	PTK2B	4.482	7.380e-06	0.453	4.560	−0.406
11	47331406	47352702	MYBPC3	6.162	7.169e-10	−1.183	6.283	1.148
11	60200270	60243137	MS4A4E	7.311	2.651 e-13	−1.438	7.730	−0.610
11	85657742	85682908	CREBZF	−5.788	7.140e-09	1.103	−5.861	−1.356
19	44890569	44903689	TOMM40	47.363	1.000e-50	10.800	47.700	−6.752
19	45178784	45216933	BLOC1S3	21.455	1.000e-50	2.760	21.200	2.017
19	45769709	45782552	DMPK	5.565	2.621e-08	0.099	5.500	1.130

* SSW meta-analysis (*q_c_* < 0.05) and MAFOCUS (*PIP* > 0.8). chromosomal location (CHR), start base position (SBP), end base position (EBP), population-stratified TWAS z-scores (Z AA, Z NHW, Z HISP).

**Table 4:** Comparison of Dense and Sparse TWAS Z-scores for Dense Model-Identified Significant Genes

Chr	Gene	NHW dense	NHW sparse	AA dense	AA sparse	HISP dense	HISP sparse

2	BIN1	4.73	4.34	2.06	2	1.86	1.6
2	GYPC	−4.43	0.06	−1.21	−1.98	−0.29	0.3
8	PTK2B	4.56	4.39	0.45	0.42	−0.41	0.15
11	MS4A4E	7.73	7.74	−1.44	−1.48	−0.61	−0.51
11	MYBPC3	6.28	6.29	−1.18	−1.18	1.15	0.96
16	COG4	4.6	3.88	0	0.29	−0.93	−0.42
19	BLOC1S3	21.2	3.47	2.76	2.16	2.02	2.53
19	DMPK	5.5	5.31	0.1	0.08	1.13	1.01
19	MARK4	10.9	11.5	−4.71	−4.45	−1.17	−2.19
19	TOMM40	47.7	48.3	10.8	10.8	−6.75	−6.88

* TWAS Z-scores comparing dense and sparse modeling approaches across three populations (Non-Hispanic White (NHW), African American (AA), and Hispanic (HISP)). Chr: Chromosome.
