# Supplementary material for: Multi-ancestry Transcriptome-Wide Association Study Reveals Shared and Population-Specific Genetic Effects in Alzheimer’s Disease
Source: bioRxiv. 2025 Nov 5:2025.11.03.686160. Preprint. [Version 1] doi: 10.1101/2025.11.03.686160 (PMC12637479; doi:10.1101/2025.11.03.686160)
Supplement: Supplement 1 [file media-1.docx]

# 1 Supplementary Material

Table S1: **Sample characteristics of three genome-wide association studies (GWAS) participants.**

| Population | AD Cases | Controls | Total |
| --- | --- | --- | --- |
| African American | 2,784 | 5,222 | 9,168 |
| Hispanic | 3,005 | 5,894 | 8,899 |
| Non-Hispanic White | 21,982 | 44,944 | 63,926 |

*African American data from Ray et al.[^31^](#ref-ExtendedGenomewideAssociation), Hispanic data from Rajabli et al.[^4^](#Xc04431740a8db6bbbc56666d5db95a113f4364c), and Non-Hispanic White data from Kunkle et al.[^32^](#X783f0451705655bebba11f075cfe61fc004513a). All summary statistics were derived from models adjusted for age, sex, and population substructure principal components.


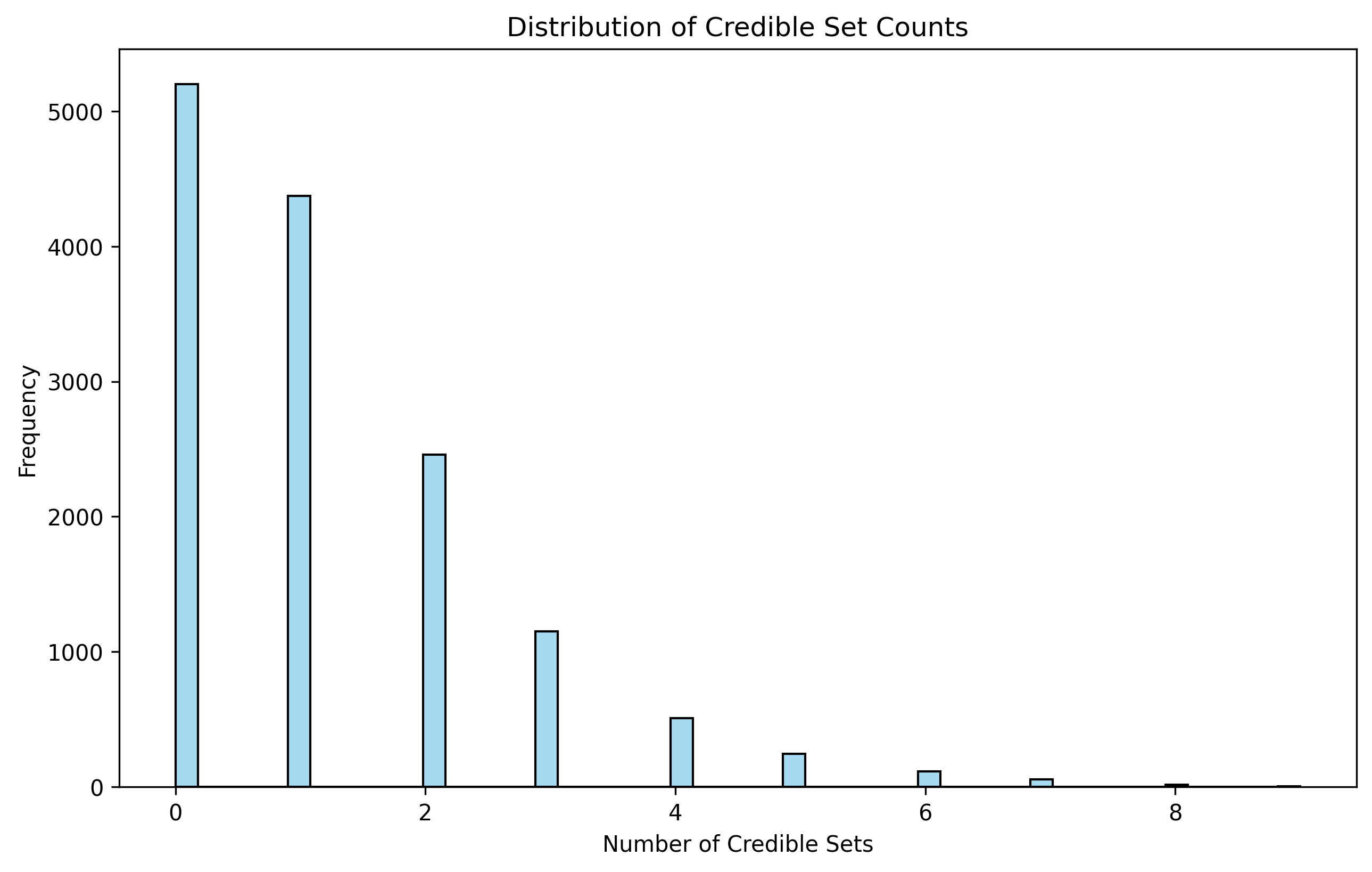


Figure S1: A histogram summarized the number of credible sets (CS) in 14,436 tested protein-coding genes. There are 8,748 of the gene has at least one CS. The mean number of CS is 1.27, and the median is 1.

Table S2: **SuShiE Fine-Mapping Parameters**

| Parameter | Type | Values |
| --- | --- | --- |
| --L | Integer | 10 |
| --pi | String | “uniform” |
| --resid-var | Float | 1e-3 |
| --effect-var | Float | 1e-3 |
| --rho | Float | 0.1 |
| --no-scale | Boolean | False |
| --no-regress | Boolean | False |
| --no-update | Boolean | False |
| --max-iter | Integer | 500 |
| --min-tol | Float | 1e-3 |
| --threshold | Float | 0.90 |
| --purity | Float | 0.5 |
| --purity_method | String | “weighted” |
| --ld-adjust | Float | 0 |
| --max-select | Integer | 250 |
| --min-snps | Integer | 100 |
| --maf | Float | 0.01 |
| --rint | Boolean | False |
| --no-reorder | Boolean | False |
| --keep-ambiguous | Boolean | False |
| --her | Boolean | True |
| --cv | Boolean | True |
| --cv-num | Integer | 5 |
| --seed | Integer | 12345 |
| --alphas | Boolean | False |

*This table provides the complete set of SuShiE fine-mapping parameters employed in our analysis that were not explicitly discussed in the main text. These include algorithm-specific parameters controlling effect estimation, optimization behavior, credible set construction, and cross-validation. All parameters are listed with the values to ensure complete reproducibility of our results.


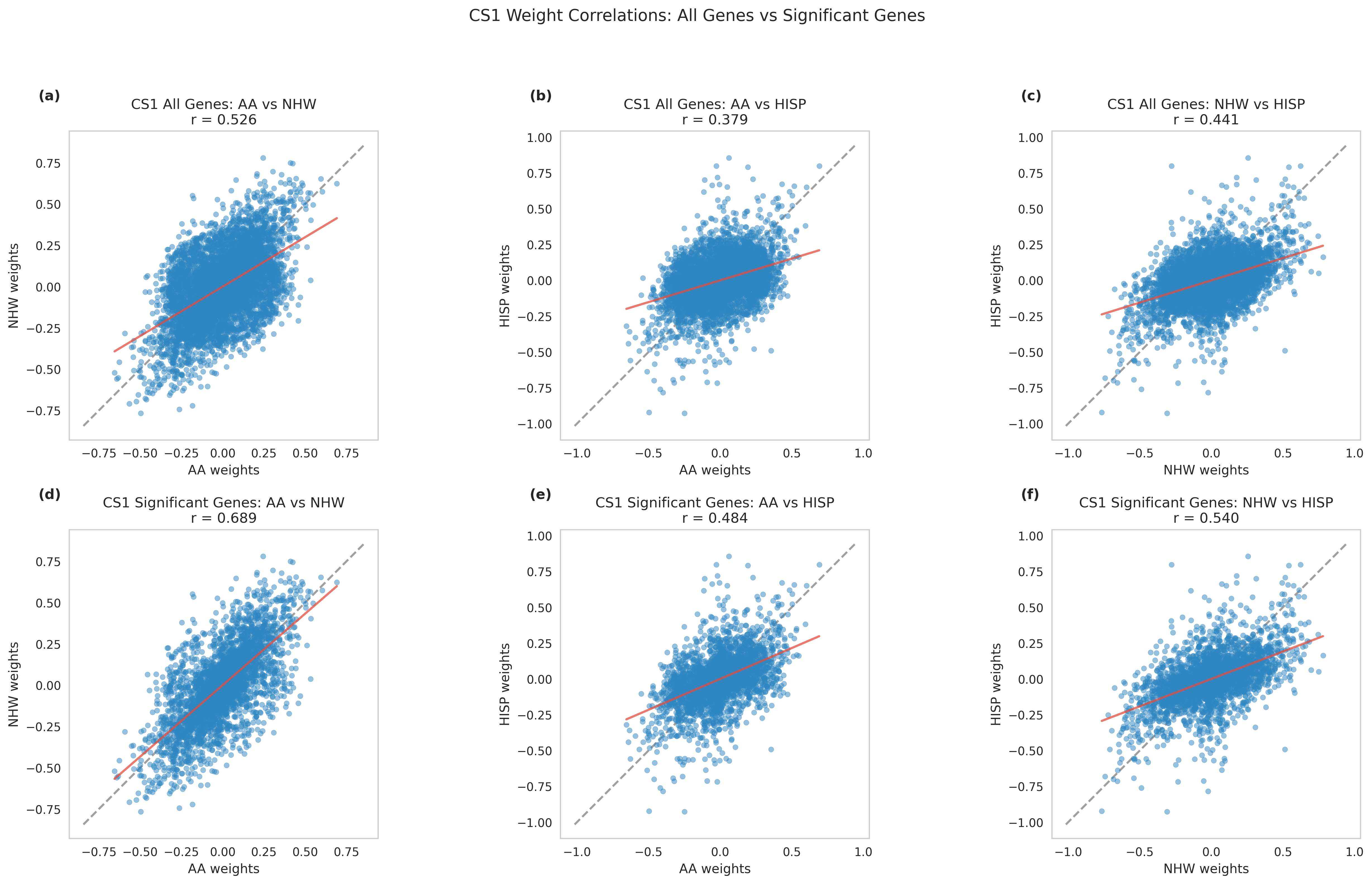


Figure S2: **Pairwise Pearson correlations of SuShiE’s population-specific posterior weights from the first credible set (CS1) across three ancestral populations (AA, NHW, and HISP).** Top row (a-c) shows correlations using all genes, while bottom row (d-f) shows correlations using only genes with significant cis-SNP heritability (P < 0.05) in at least one population. (a,d) Comparison between AA and NHW populations. (b,e) Comparison between AA and HISP populations. (c,f) Comparison between NHW and HISP populations. Gray dashed lines represent the diagonal (y=x), and red solid lines show the best-fit linear regression. Pearson correlation coefficients (r) are shown for each comparison. Higher correlations are observed in significant genes compared to all genes, with the strongest correlation between AA and NHW populations (r=0.689 for significant genes)


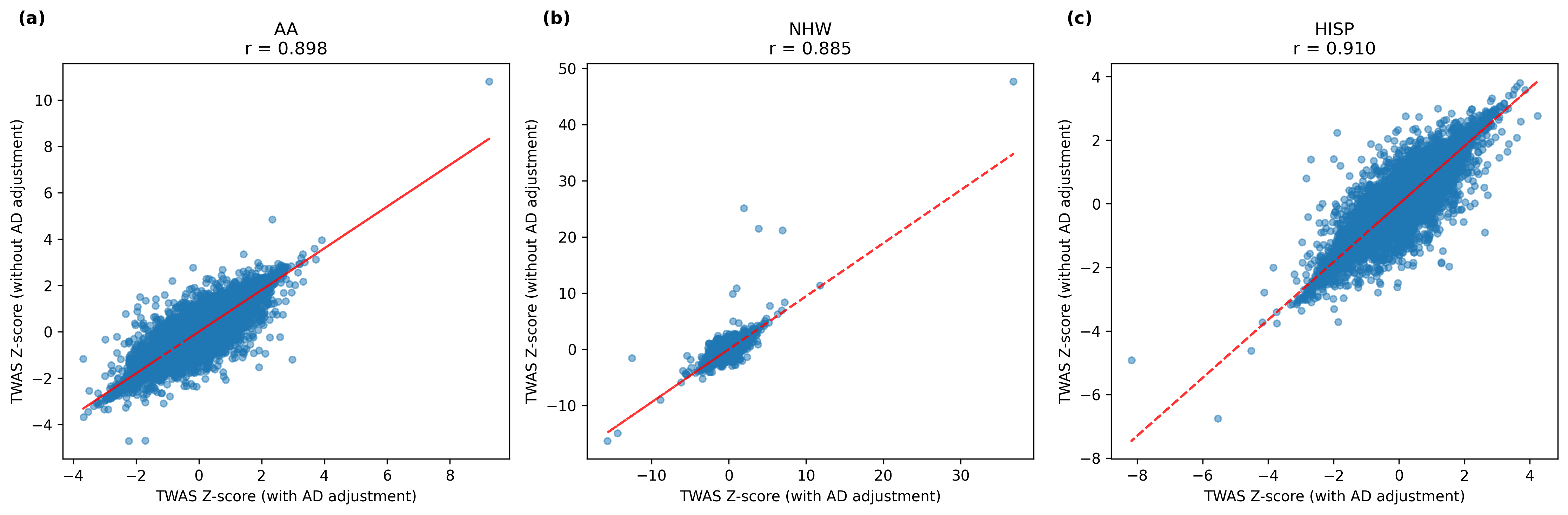


Figure S3: **Correlation of TWAS Z-scores between models with and without Alzheimer’s disease (AD) adjustment across three ancestral populations.** Scatter plots showing the relationship between TWAS Z-scores from models with AD adjustment (x-axis) and without AD adjustment (y-axis) for (a) African American (AA), (b) Non-Hispanic White (NHW), and (c) Hispanic (HISP) populations. Each point represents a gene, and the red line indicates the best-fit linear regression. Pearson correlation coefficients (r) demonstrate strong concordance between the two models across all populations (AA: r = 0.898, NHW: r = 0.885, HISP: r = 0.910), suggesting that AD adjustment has minimal impact on overall TWAS association patterns while preserving population-specific expression patterns.


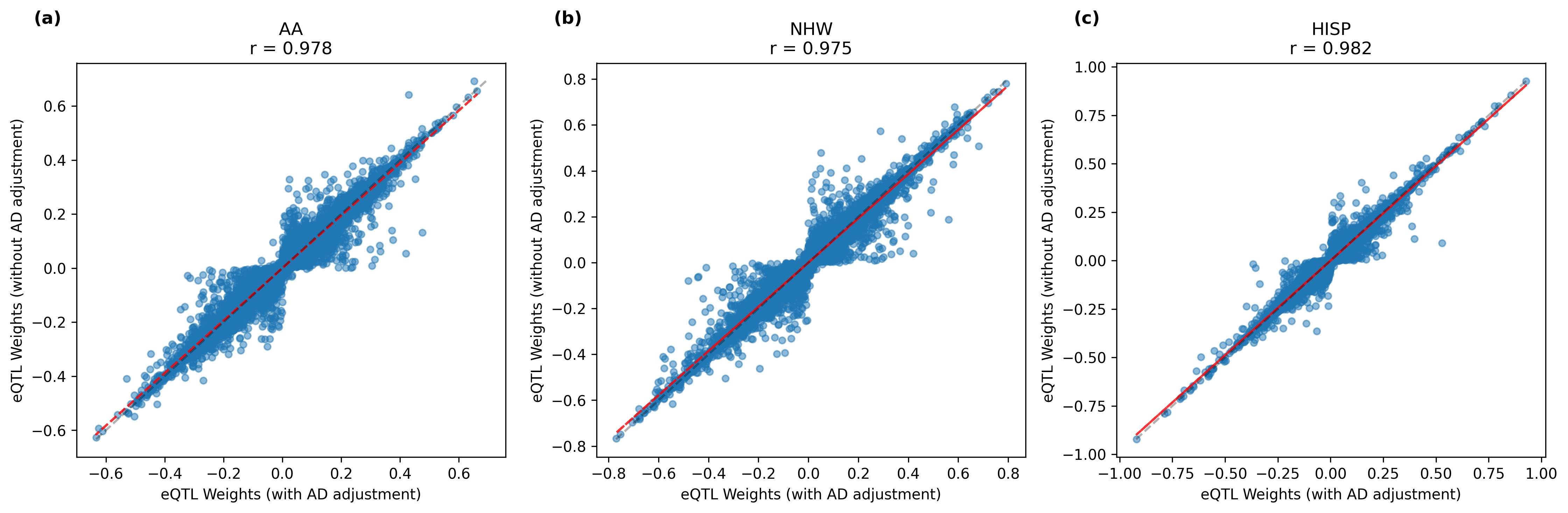


Figure S4: **Comparison of eQTL weights between models with and without Alzheimer’s disease (AD) adjustment across three ancestral populations.** Scatter plots displaying the correlation between eQTL weights from models with AD adjustment (x-axis) and without AD adjustment (y-axis) for (a) African American (AA), (b) Non-Hispanic White (NHW), and (c) Hispanic (HISP) populations. Each point represents an individual SNP-gene pair in credible sets. The red dashed lines indicate the best-fit linear regression. Extremely high Pearson correlation coefficients (AA: r = 0.978, NHW: r = 0.975, HISP: r = 0.982) demonstrate that genetic effect estimates remain highly consistent regardless of AD adjustment, suggesting that the underlying genetic architecture of gene expression is robustly captured by both modeling approaches across diverse populations.

Table S3: **Comparison of SNP inclusion in credible sets between models with and without Alzheimer’s disease adjustment.**

| Credible Set | Total SNPs (AD-adjusted) | Total SNPs (Unadjusted) | Shared SNPs | Shared % (AD-adjusted) | Shared % (Unadjusted) |
| --- | --- | --- | --- | --- | --- |
| CS1 | 88,675 | 89,173 | 67,267 | 75.86% | 75.43% |
| CS2 | 44,556 | 43,302 | 23,583 | 52.93% | 54.46% |
| CS3 | 23,057 | 21,691 | 9,610 | 41.68% | 44.30% |
| Overall | 177,307 | 173,990 | 127,718 | 72.03% | 73.41% |

*This table presents the overlap of SNPs identified in credible sets from SuShiE with and without adjustment for Alzheimer’s disease (AD). For each credible set (CS1-CS3, in decreasing order of importance), the table shows the total number of SNPs in each model, the number of shared SNPs between models, and the percentage of SNPs shared. The “Overall” row summarizes statistics across all credible sets. While the highest-ranked credible set (CS1) shows substantial concordance (~75% overlap), the agreement decreases for lower-ranked credible sets, suggesting that AD adjustment has a more pronounced effect on secondary genetic signals.

Previous studies[^40^](#ref-siebertsLargeEQTLMetaanalysis2020)^–^[^42^](#ref-ngXQTLMapIntegrates2017) have demonstrated that cis-eQTL effects are largely consistent between disease and control cohorts and healthy individual cohorts, particularly in neuropsychiatric and neurodegenerative disorders, with genetic regulation of gene expression remaining stable across disease states. This same consistency was observed in the MAGENTA study for eQTL mapping and TWAS analysis. By not adjusting for AD status in our eQTL models, we preserved potential disease-related effects on expression that might be relevant to AD pathogenesis. Our sensitivity analyses comparing AD-adjusted and unadjusted models confirmed this approach, showing high concordance in TWAS associations across all populations ($r\approx0.9$) (Figure [S3](#fig:compare_AD_status_TWAS_assoc)). We observed over 73% overlapping SNPs in credible sets, with the first credible sets, which capture primary regulatory effects, showing particularly high overlap at 75% shared eQTLs (Table [S3](#tbl:AD_adj_overlap_SNP)). The effect size correlations for overlapped eQTLs were remarkably high ($r\approx0.98$) (Figure [S4](#fig:compare_AD_status_eqtl_weight_assoc)), and none of the significant genes identified in the main analysis showed significant changes in TWAS Z-scores scores after adjusting gene expression prediction models for AD status. These findings demonstrate that while preserving disease-related expression patterns, the core genetic regulatory mechanisms remain robust and reliable for the TWAS analysis.

Table S4: **Credible set analysis of 10 AD-related genes analyzed in sparse models**

| Gene | Credible Set | # Variants | Min PIP | Median PIP | Max PIP | Sum PIP |
| --- | --- | --- | --- | --- | --- | --- |
| BIN1 | 1 | 1 | 1.000 | 1.000 | 1.000 | 1.000 |
| BIN1 | 2 | 2 | 0.499 | 0.499 | 0.499 | 0.999 |
| BLOC1S3 | 1 | 10 | 0.018 | 0.124 | 0.124 | 0.917 |
| COG4 | 1 | 4 | 0.069 | 0.222 | 0.395 | 0.908 |
| COG4 | 2 | 5 | 0.023 | 0.054 | 0.709 | 0.921 |
| DMPK | 1 | 1 | 0.999 | 0.999 | 0.999 | 0.999 |
| DMPK | 2 | 8 | 0.086 | 0.090 | 0.185 | 0.941 |
| DMPK | 3 | 3 | 0.175 | 0.243 | 0.559 | 0.976 |
| DMPK | 4 | 7 | 0.043 | 0.066 | 0.401 | 0.927 |
| DMPK | 5 | 52 | 0.007 | 0.020 | 0.037 | 0.901 |
| DMPK | 6 | 11 | 0.013 | 0.085 | 0.160 | 0.903 |
| GYPC | 1 | 3 | 0.332 | 0.332 | 0.332 | 0.997 |
| MARK4 | 1 | 2 | 0.193 | 0.459 | 0.725 | 0.918 |
| MARK4 | 2 | 1 | 0.979 | 0.979 | 0.979 | 0.979 |
| MARK4 | 3 | 1 | 0.979 | 0.979 | 0.979 | 0.979 |
| MARK4 | 4 | 5 | 0.083 | 0.212 | 0.212 | 0.930 |
| MARK4 | 5 | 4 | 0.034 | 0.212 | 0.472 | 0.930 |
| MS4A4E | 1 | 3 | 0.084 | 0.283 | 0.559 | 0.926 |
| MS4A4E | 2 | 6 | 0.037 | 0.139 | 0.269 | 0.931 |
| MYBPC3 | 1 | 34 | 0.008 | 0.022 | 0.116 | 0.907 |
| PTK2B | 1 | 6 | 0.059 | 0.171 | 0.238 | 0.903 |
| PTK2B | 2 | 9 | 0.078 | 0.089 | 0.166 | 0.932 |
| PTK2B | 3 | 12 | 0.012 | 0.036 | 0.474 | 0.904 |
| TOMM40 | 1 | 1 | 0.998 | 0.998 | 0.998 | 0.998 |
| TOMM40 | 2 | 2 | 0.382 | 0.497 | 0.613 | 0.994 |
| TOMM40 | 3 | 1 | 0.950 | 0.950 | 0.950 | 0.950 |
| TOMM40 | 4 | 9 | 0.005 | 0.034 | 0.352 | 0.903 |
| TOMM40 | 5 | 15 | 0.011 | 0.023 | 0.410 | 0.911 |

*Summary of fine-mapping results for AD-associated genes (complementary to locus plots of 10 significant genes analyzed in sparse models). The Min/Median/Max PIP columns show the distribution of posterior inclusion probabilities within each set, while Sum PIP indicates the cumulative PIP captured by the credible set.

Table S5: **Cross-population cis-heritability and predictive performance of expression models**

| Gene | h²_AA | R²_AA | h²_NHW | R²_NHW | h²_HISP | R²_HISP |
| --- | --- | --- | --- | --- | --- | --- |
| BIN1 | 0.0312* | 0.1787*** | 0.0562*** | 0.2626*** | 0.0338* | 0.1165*** |
| TOMM40 | 0.0000 | -0.0015 | 0.0448** | 0.1326*** | 0.0408 | -0.0010 |
| DMPK | 0.0824* | 0.1459*** | 0.0158 | 0.0683*** | 0.0274 | 0.0259** |
| PTK2B | 0.0200 | 0.0576*** | 0.0154 | 0.1197*** | 0.0284 | 0.0399*** |
| MYBPC3 | 0.0080 | 0.0181* | 0.0520*** | 0.1683*** | 0.0250 | 0.0013 |
| GYPC | 0.0000 | -0.0038 | 0.0000 | -0.0042 | 0.0169* | 0.0163* |
| CREBZF | 0.0020 | -0.0043 | 0.0004 | -0.0025 | 0.0111 | 0.0013 |
| BLOC1S3 | 0.0000 | -0.0041 | 0.0483** | 0.0312** | 0.0164 | 0.0055 |
| MS4A4E | 0.0173 | 0.0501*** | 0.0002 | 0.0004 | 0.0000 | -0.0032 |
| COG4 | 0.0646*** | 0.1227*** | 0.0193* | 0.0487*** | 0.0000 | 0.0112* |
| MARK4 | 0.0000 | 0.0033 | 0.1373*** | 0.3079*** | 0.0358 | 0.1017*** |

*Per-gene cis-SNP heritability (h²) and 5-fold cross-validated prediction R² (R²) reported by population (AA, NHW, HISP). Asterisks denote nominal two-sided significance for the corresponding entry (*** $P<0.001$, ** $P<0.01$, * $P<0.05$; no symbol = not significant). Values are shown to four decimals; significance is evaluated on unrounded P-values.

Table S6: **MAFOCUS PIP of significant TWAS associations**

| gene_symbol | PIP_NHW | PIP_AA | PIP_HISP | PIP_ME |
| --- | --- | --- | --- | --- |
| TOMM40 | 1.000 | 1.000 | 1.000 | 1.000 |
| BIN1 | 1.000 | 0.857 | 0.140 | 1.000 |
| GYPC | 1.000 | 0.178 | 0.149 | 1.000 |
| BLOC1S3 | 1.000 | 0.006 | 0.029 | 1.000 |
| MS4A4E | 0.995 | 0.002 | 0.001 | 0.998 |
| MYBPC3 | 0.990 | 0.004 | 0.008 | 0.996 |
| CREBZF | 1.000 | 0.011 | 0.017 | 0.972 |
| DMPK | 0.863 | 0.003 | 0.004 | 0.916 |
| GARIN2 | 0.030 | 0.039 | 0.908 | 0.883 |
| PTK2B | 0.978 | 0.027 | 0.025 | 0.866 |
| CEBPZOS | 0.928 | 0.011 | 0.013 | 0.596 |
| COG4 | 0.969 | 0.003 | 0.004 | 0.418 |
| CCDC9 | 0.855 | 0.003 | 0.003 | 0.245 |
| MARK4 | 0.130 | 0.955 | 0.005 | 0.020 |

*Dense-model TWAS genes significant by both MAFocus (PIP $\geq$ 0.8) and FUSION TWAS ($q_{c}<0.05$), showing the maximum PIPfor NHW, AA, HISP, and cross-population (ME).

Table S7: **Validation of TWAS associations using eQTL reference panels from TOPMed MESA visit 1 by population**

| Gene | Z AA | Z NHW | Z HISP |
| --- | --- | --- | --- |
| BIN1 | 2.18* | 2.62** | 0.99 |
| TOMM40 | 9.57*** | 15.30*** | -3.64*** |
| DMPK | 0.28 | 4.28*** | 2.96** |
| PTK2B | 0.00 | 4.49*** | 0.38 |
| MYBPC3 | -0.06 | 5.78*** | 0.60 |
| GYPC | 0.78 | -0.93 | 0.66 |
| CREBZF | 1.20 | -1.04 | -0.92 |
| BLOC1S3 | -0.17 | -4.43*** | 0.01 |
| MS4A4E | 0.91 | 6.72*** | -1.97* |
| COG4 | -0.18 | 0.28 | 0.76 |
| MARK4 | -1.66 | 0.51 | -1.17 |

*Per-gene TWAS Z-scores from the TOPMed MESA visit 1 validation dataset, stratified by population (AA, NHW, HISP). Asterisks denote nominal two-sided significance for the corresponding entry (*** $P<0.001$, ** $P<0.01$, * $P<0.05$; no symbol = not significant).


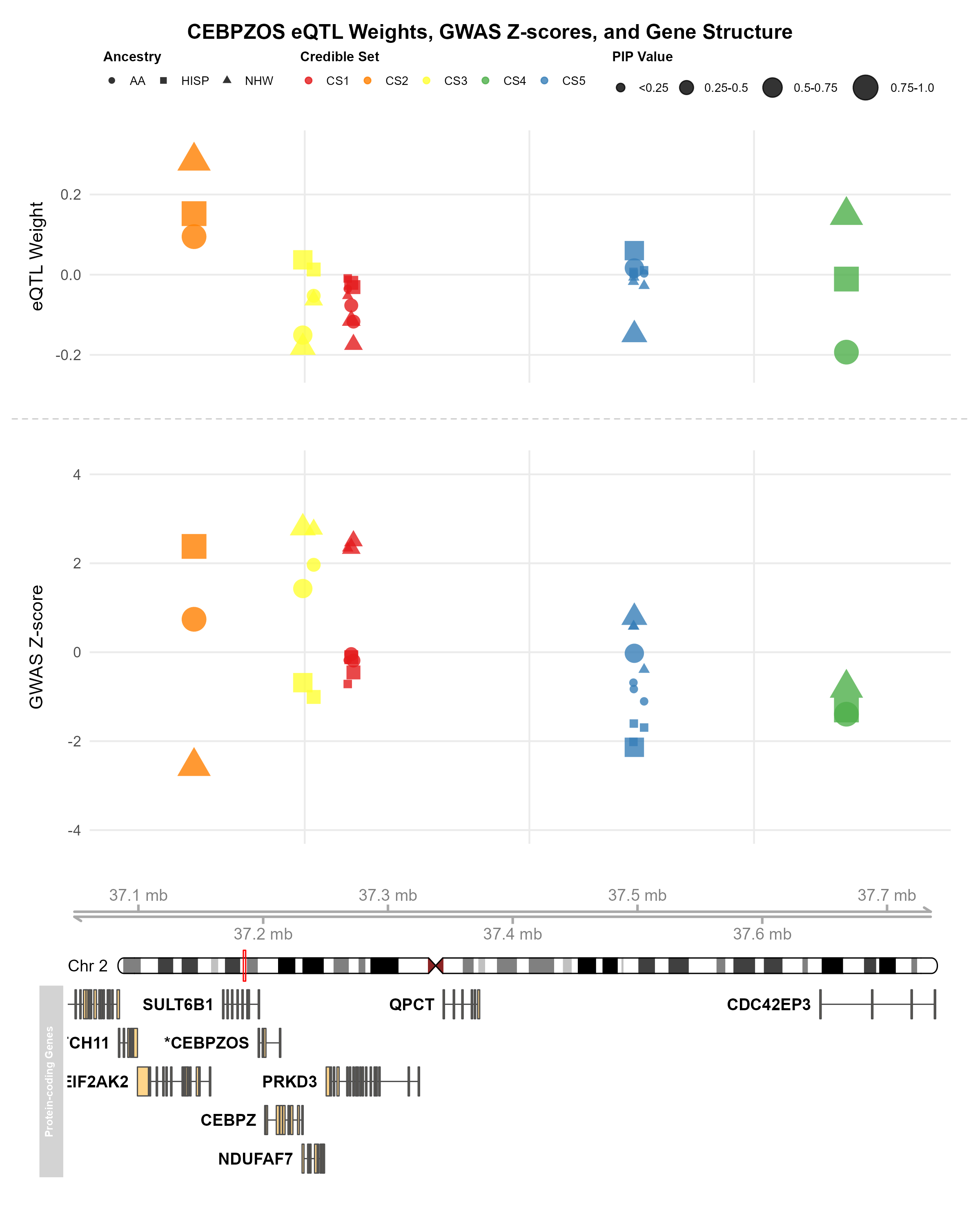


Figure S5: **Fine-mapped eQTLs and GWAS associations for the CEBPZOS locus.** The figure displays the relationship between eQTL weights (top panel), GWAS Z-scores (middle panel), and gene structure (bottom panel) for CEBPZOS on chromosome 2. In the top and middle panels, eQTL weights and GWAS associations are shown across three ancestral populations (African American (AA, circles), Hispanic (HISP, squares), and Non-Hispanic White (NHW, triangles)), with variants colored by credible set membership. Point sizes indicate posterior inclusion probability (PIP) values. The bottom panel illustrates the genomic structure of CEBPZOS at chromosome 2.


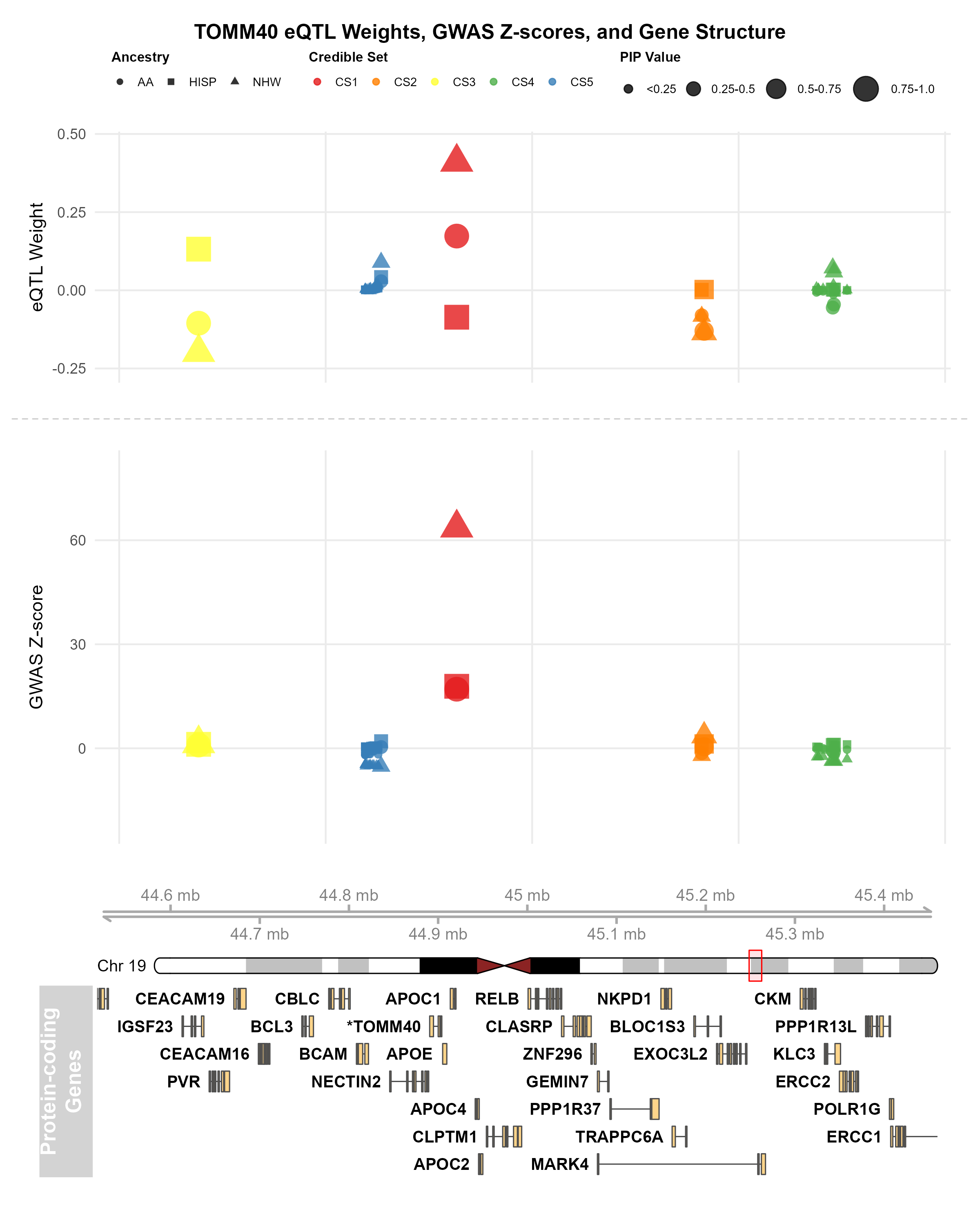


Figure S6: **Fine-mapped eQTLs and GWAS associations for the TOMM40 locus.** The figure displays the relationship between eQTL weights (top panel), GWAS Z-scores (middle panel), and gene structure (bottom panel) for TOMM40 on chromosome 19. In the top and middle panels, eQTL weights and GWAS associations are shown across three ancestral populations (African American (AA, circles), Hispanic (HISP, squares), and Non-Hispanic White (NHW, triangles)), with variants colored by credible set membership. Point sizes indicate posterior inclusion probability (PIP) values. The bottom panel illustrates the genomic structure of TOMM40 at chromosome 19.

According to Figure [S6](#fig:tomm40_locus), *TOMM40*’s variant in the first credible set exhibited heterogeneous effects across populations. Notably, this variant rs429358 (chr19:44908684_T_C), which corresponds to the APOE-ε4 allele, showed distinct population-specific patterns. In the Hispanic population, this variant demonstrated negative eQTL weights, while in NHW and AA populations, the weights were positive. This population-specific regulatory pattern likely explains the opposite TWAS associations observed in the Hispanic population compared to NHW and AA. The result reveals that while the GWAS signals are consistently strong across all populations in this region, the direction of gene expression regulation differs substantially in Hispanic individuals. When the ε4 allele was removed from the prediction model, the associations were significantly diminished in all three populations ($Z_{NHW}=-5.55$; $Z_{AA}=0.22$; $Z_{HISP}=1.63$). Our findings suggest that in addition to its direct protein-coding effects, ε4-defining variants may also influence expression of *TOMM40* in whole-blood. Overall, this finding suggests that the underlying regulatory mechanisms affecting *TOMM40* expression may be driven by the APOE effect.


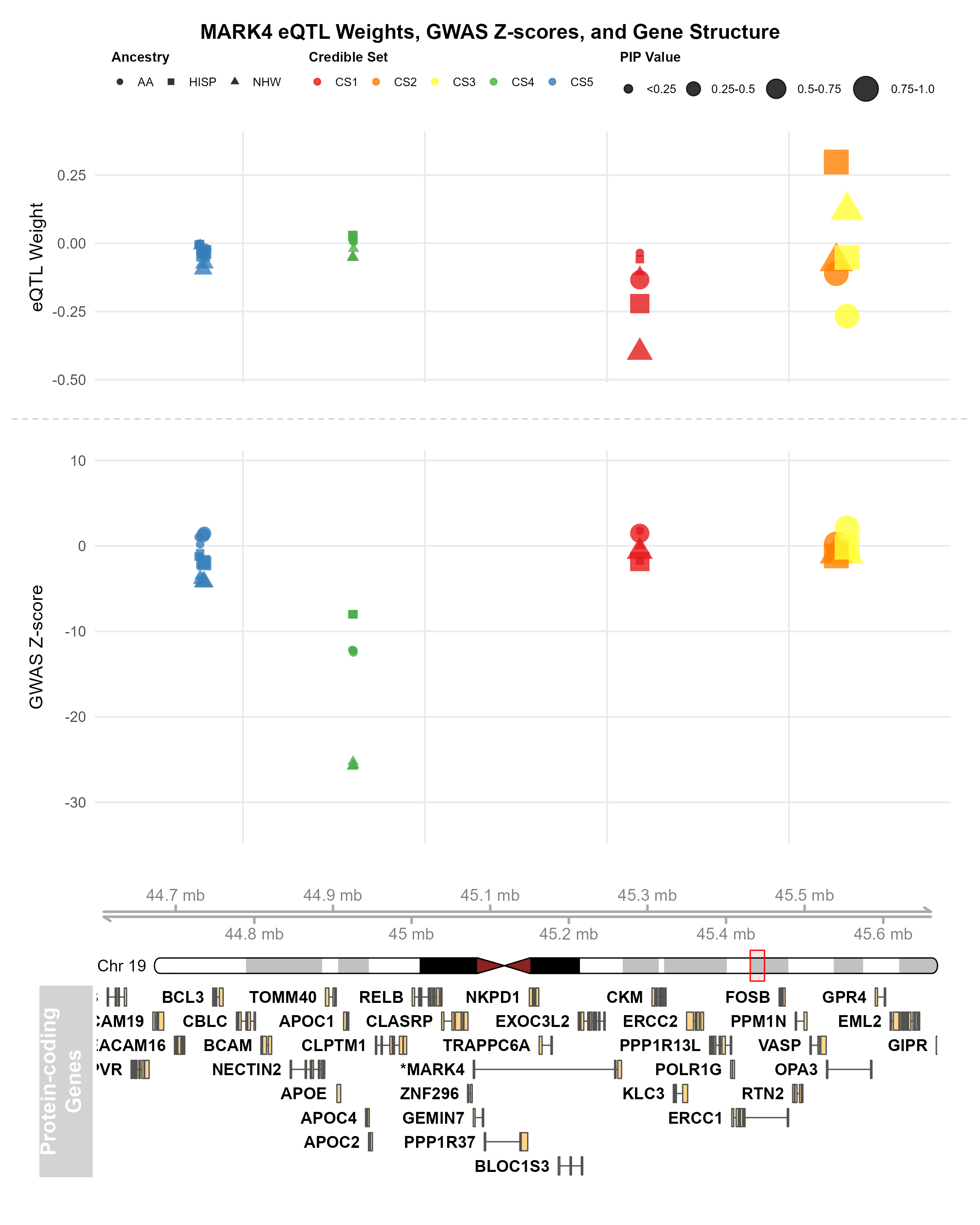


Figure S7: **Fine-mapped eQTLs and GWAS associations for the MARK4 locus.** The figure displays the relationship between eQTL weights (top panel), GWAS Z-scores (middle panel), and gene structure (bottom panel) for MARK4 on chromosome 19. In the top and middle panels, eQTL weights and GWAS associations are shown across three ancestral populations (African American (AA, circles), Hispanic (HISP, squares), and Non-Hispanic White (NHW, triangles)), with variants colored by credible set membership. Point sizes indicate posterior inclusion probability (PIP) values. The bottom panel illustrates the genomic structure of MARK4 at chromosome 19.

Table S8: **COG4 credible set variant frequencies across ancestries**

| SNP ID | CS | NHW Freq | AA Freq | HISP Freq |
| --- | --- | --- | --- | --- |
| chr16:70508818_C_T | 1 | 0.034 | 0.241 | 0.036 |
| chr16:70616971_G_A | 1 | 0.038 | 0.228 | 0.045 |
| chr16:70617075_T_C | 1 | 0.038 | 0.228 | 0.045 |
| chr16:70470982_T_C | 1 | 0.034 | 0.248 | 0.045 |
| chr16:70666311_A_C | 2 | 0.496 | 0.174 | 0.380 |
| chr16:70698580_G_A | 2 | 0.521 | 0.143 | 0.445 |
| chr16:70696670_A_C | 2 | 0.521 | 0.143 | 0.447 |
| chr16:70690022_A_G | 2 | 0.575 | 0.413 | 0.505 |
| chr16:70669915_T_G | 2 | 0.466 | 0.179 | 0.435 |

*SNP ID uses chr{chr}:pos_ref_alt. CS is the SuShiE credible set index. NHW/AA/HISP Freq are alternative allele frequencies from each group.


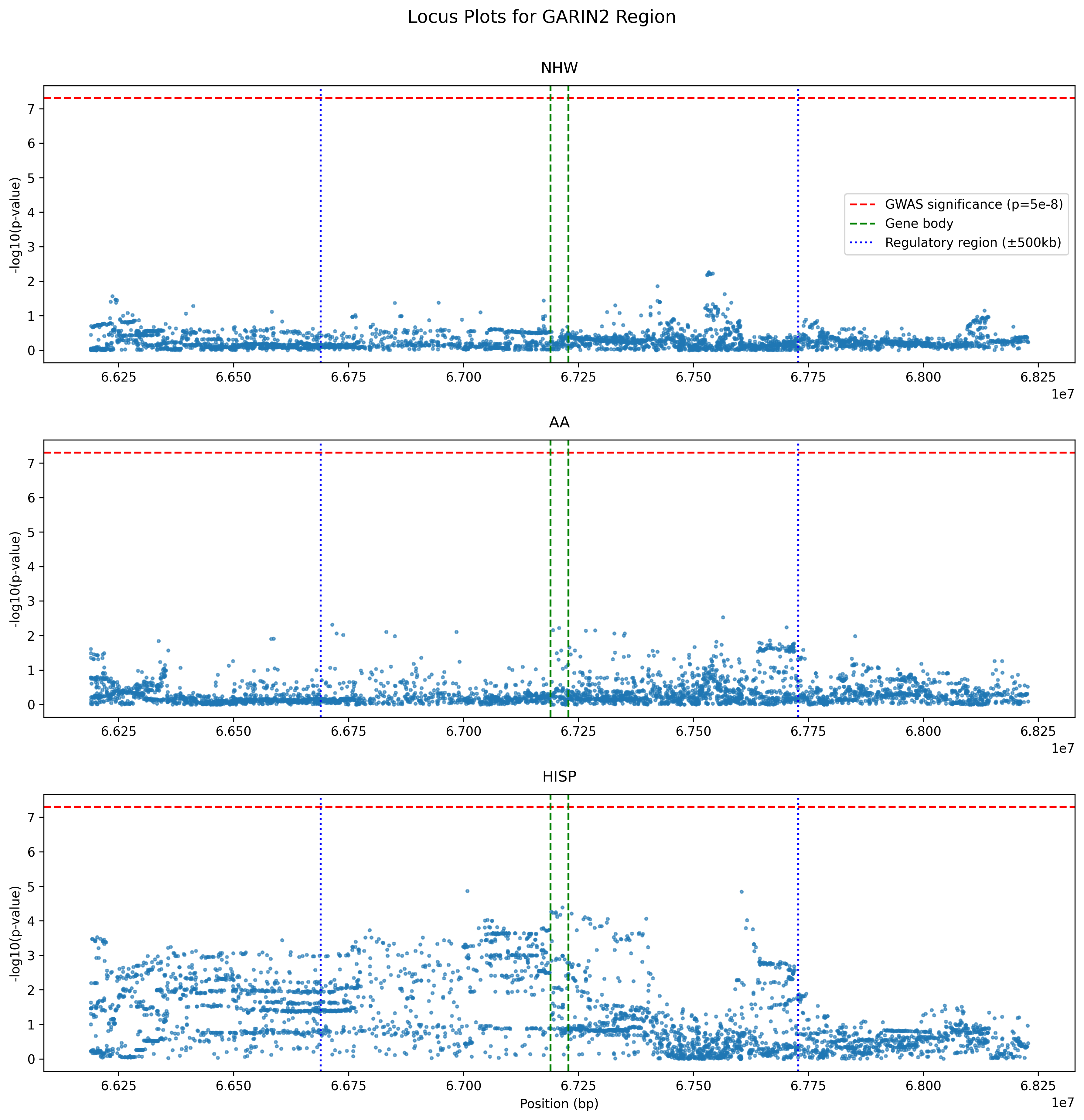


Figure S8: **GWAS associations of cis-regulatory variants near GARIN2 showed suggestive significance in HISP AD GWAS only** The locus plots showed the GWAS associations’ $-{log}_{10}\left( P \right)$ for NHW, AA, and HISP population respectively. HISP showed uniform suggestive significance ($P<1*{10}^{-3}$).
